# A Causal Model of Ion Interference Enables Assessment and Correction of Ratio Compression in Multiplex Proteomics

**DOI:** 10.1016/j.mcpro.2023.100694

**Published:** 2023-12-12

**Authors:** Moritz Madern, Wolfgang Reiter, Florian Stanek, Natascha Hartl, Karl Mechtler, Markus Hartl

**Affiliations:** 1 Max Perutz Labs, Mass Spectrometry Facility, Vienna Biocenter Campus (VBC), Vienna, Austria; 2 Department for Biochemistry and Cell Biology, Center for Molecular Biology, University of Vienna, Vienna Biocenter Campus (VBC), Vienna, Austria; 3 Research Institute of Molecular Pathology (IMP), Vienna Biocenter Campus (VBC), Vienna, Austria

**Keywords:** isobaric label, TMT, ion interference, ratio compression

## Abstract

Multiplex proteomics using isobaric labeling tags has emerged as a powerful tool for the simultaneous relative quantification of peptides and proteins across multiple experimental conditions. However, the quantitative accuracy of the approach is largely compromised by ion interference, a phenomenon that causes fold changes to appear compressed. The degree of compression is generally unknown, and the contributing factors are poorly understood. In this study, we thoroughly characterized ion interference at the MS2 level using a defined two-proteome experimental system with known ground-truth. We discovered remarkably poor agreement between the apparent precursor purity in the isolation window and the actual level of observed reporter ion interference in MS2 scans—a discrepancy that we found resolved by considering cofragmentation of peptide ions hidden within the spectral “noise” of the MS1 isolation window. To address this issue, we developed a regression modeling strategy to accurately predict reporter ion interference in any dataset. Finally, we demonstrate the utility of our procedure for improved fold change estimation and unbiased PTM site-to-protein normalization. All computational tools and code required to apply this method to any MS2 TMT dataset are documented and freely available.

## Highlights

• Ion interference in multiplex proteomics can be modeled *via* linear regression.
• Spectral noise is crucial in explaining ion interference at MS2 level.
• Model-based interference prediction offers solutions to ratio compression.
• Interference needs to be considered for unbiased PTM site-to-protein normalization.

## Graphical Abstract

**Figure:**
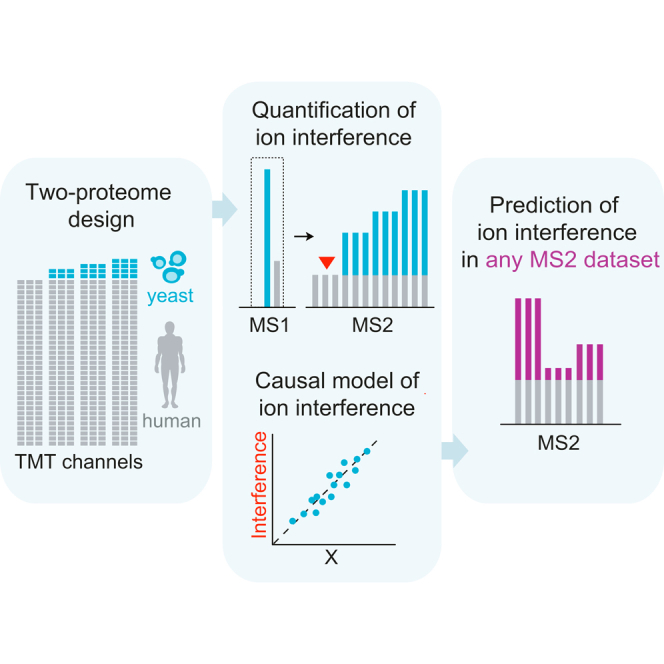

## In Brief

Using a defined two-proteome experimental setup, we thoroughly characterized ion interference. Exploration of the underlying causal mechanism revealed that noise at MS1 level is pivotal in explaining reporter ion interference at MS2 level. New insights into reporter ion generation were combined to develop a regression model capable of predicting the degree of ion interference in any MS2-quantified dataset. This modeling strategy offers novel computational solutions to address the ratio compression problem in multiplex proteomics.

In mass spectrometry (MS)-based proteomics, isobaric labeling systems such as tandem mass tags (TMT) have been established to efficiently quantify proteins across multiple conditions within a single experiment (1, 2, 3). Their high throughput is based on the ability to combine up to 18 samples in one experiment (4), using sample-specific isobaric labels that allow joint processing and measurement of peptides. Upon fragmentation of the labels in the mass spectrometer, sample-specific reporter ions are generated (1), revealing the relative abundance of peptides and proteins for each sample. As all samples are measured at the same time, the approach is largely free of missing values. Further, more complex sample processing steps like offline-fractionation (2) or the enrichment for posttranslational modifications (PTMs) (2, 3) benefit from the early pooling of samples by eliminating additional technical variation.

However, despite the elegance in design, multiplex proteomics struggles with accurate relative quantification due to the phenomenon termed ion interference. Concomitant fragmentation of targeted precursor ions along with interfering nonprecursor ions in the same isolation window results in convoluted reporter ion profiles (5, 6). As the majority of proteins in a typical large-scale proteomic experiment are nondifferentially expressed, relative quantitative differences of precursor peptides become suppressed by a uniform background of interfering reporter ion signal. This effect is also referred to as “ratio compression” (6). Extensive sample fractionation and decreased isolation window widths help to reduce this effect (7, 8, 9, 10), yet they do not completely remove it. Interestingly, despite ratio compression, the ability to distinguish differentially from nondifferentially expressed proteins using isobaric labeling-based quantification is comparable or even outperforms label-free quantification due to increased quantitative precision and data completeness within the same multiplex set (11). However, compressed fold changes make it more difficult to interpret results in a biologically meaningful way. Therefore, different strategies have been developed to address and mitigate the ratio compression problem.

Promising technical solutions encompass gas-phase purification (12), dual isolation width acquisition (9), high-field asymmetric-waveform ion mobility MS (FAIMS) (13), and MS3-based quantification (10, 14, 15, 16). Unfortunately, these methods come with their own set of limitations as they require specialized instrumentation for data acquisition (10, 12, 13, 14, 15), and the measurement of additional scans per precursor generally decreases measurement speed and thus the number of IDs (9, 10, 14, 15, 16). New isobaric tags that generate interference-free reporter ions have been developed by Winter *et al*. (17) but they are commercially not yet available, preventing wider testing and adoption by other labs. Wühr *et al.* described an alternative quantification method in which the complementary fragment ion cluster in the MS2 spectrum is used for the quantitative profiling (18, 19). Unfortunately, most current MS instruments lack the required resolution to separate heavy N and C isotope mass differences in the higher m/z region of the complementary ions, which reduces the multiplexing capabilities to a maximum of nine channels (4, 20). Another hurdle of quantification *via* the complementary ion cluster lies in the complementary ion formation itself, as currently available isobaric labeling systems are not optimized to generate complementary fragment ions (21). A recent study examined the use of combined 11plex and 18plex TMT reagents to estimate interference levels (22). These estimates successfully enabled the decompression of fold changes; however, the general applicability of this innovative multiplexing strategy needs to be further evaluated.

Other strategies address ratio compression *in silico*. Savitski *et al.* proposed an interference correction algorithm (23) based on the precursor ion purity in the MS1 isolation window range (24). Niu *et al.* estimated the level of interference *via* Lys-y_1_ and Arg-y_1_ ion intensities in MS2 scans, ascribing them to either target or interfering peptides (8). Other approaches attempt to correct ratio compression by modeling the quantitative relationship between observed and theoretical fold changes provided by a synthetic peptide spike-in (6, 25). Similarly, the modeling of observed fold changes in dependence of precursor ion purities and signal to noise ratios for different peptides of the same protein has been proposed (26). Altogether, we believe that these strategies have certain limitations due to their phenomenological approach in addressing the interference issue. They rely on correlations between ion interference and a single measured variable, providing only a partial explanation for the extent of ratio compression in an experiment. This simplified perspective can be attributed to the current limitations in our understanding of the causal factors underlying interference. Consequently, the proposed computational methods are not fully equipped to address ratio compression at the level of individual peptide spectrum matches (PSMs), thereby restricting their effectiveness in mitigating ratio compression throughout an entire experiment.

In this study, we aimed to address ratio compression by building on a holistic mechanistic understanding of the underlying phenomenon itself, that is, ion interference. To this end, we designed a controlled two-proteome experiment which allowed us to directly observe reporter ion interference (here defined as the reporter ion signal at MS2 level caused by ion interference) for a substantial fraction of peptides while simultaneously monitoring the accompanied ratio compression effect on known theoretical fold changes. Taking advantage of this design, we first explored the dependence of ion interference on varying measurement parameters and the accompanying effect on differential expression (DE) analysis. We then aimed to causally understand the measured reporter ion interference by means of multiple linear regression modeling. Supporting the findings of previous work by Savitski *et al.* (23), we found that the degree of interference at MS2 level is only duly explained when accounting for spectral “noise” at the MS1 level, as the apparent precursor ion purity in the isolation window generally underestimates the true extent of reporter ion interference. By generalizing such a model, we found a way to accurately predict the degree reporter ion interference in any MS2-quantified multiplex proteomics dataset. Finally, we demonstrate how this gain in information can be used to decompress fold changes affected by ratio compression and carry out normalization of PTM sites to underlying protein abundances unbiased by differences in ion interference.

## Experimental Procedures

### Experimental Design and Statistical Rationale

**Fig. 1:**
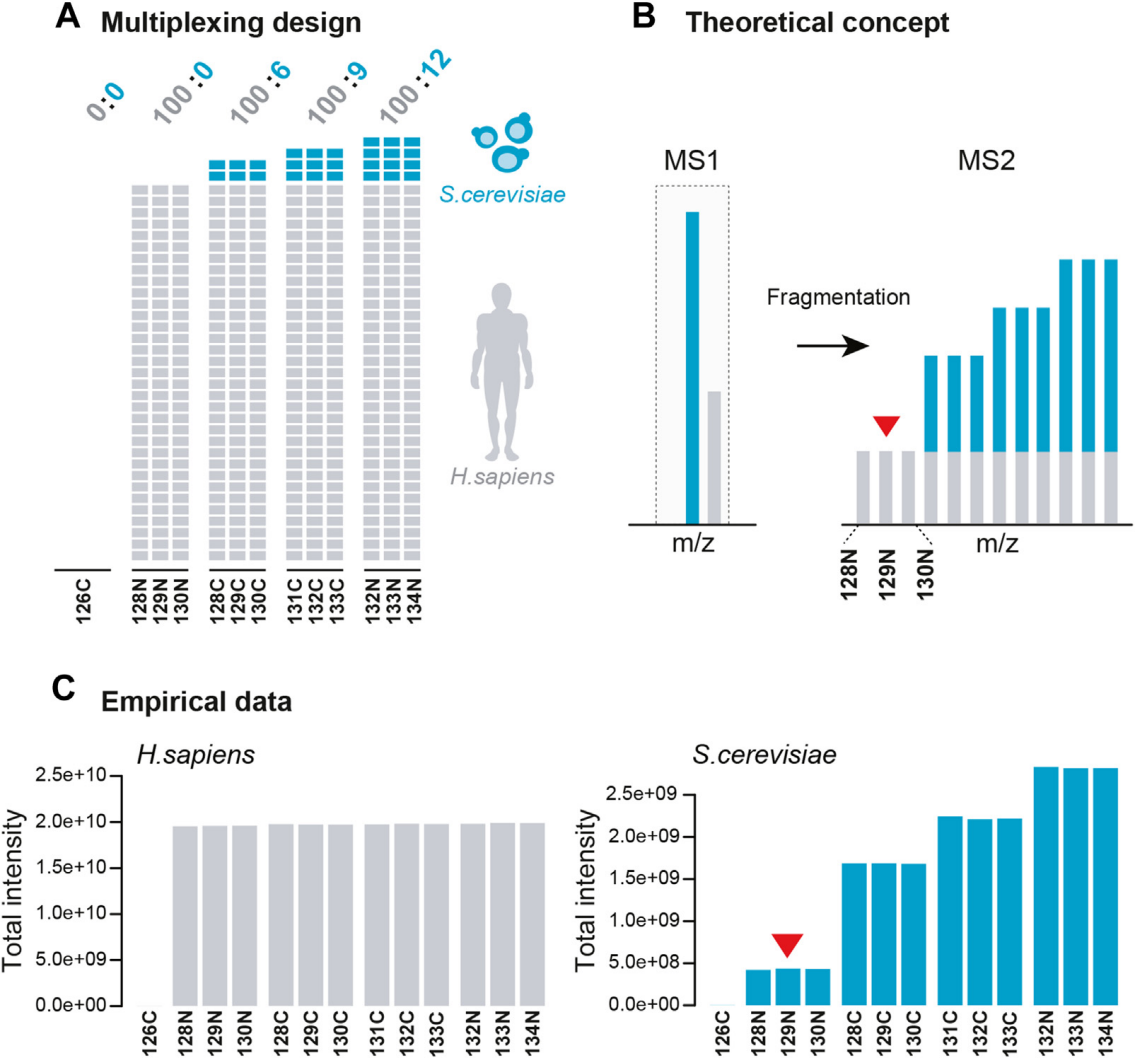
**Artificial two-proteome dataset for exploring ion interference.***A*, schematic depiction of sample preparation and TMTpro 16plex labeling scheme. Samples were defined mixtures of *Homo sapiens* Jurkat E6-1 (*gray*) and *Saccharomyces cerevisiae* (*turquoise*) peptides from whole-proteome digests. Relative mass abundance ratios (w/w) of human to yeast are displayed on *top* of each sample group. *B*, concept of measuring reporter ion interference at the MS2 level *via* channels 128N, 129N, and 130N (marked by *red arrow*) for a targeted yeast peptide. *C*, total measured channel intensities of quantified human (*left*) and yeast (*right*) peptides after between-sample normalization. The *red arrow* marks human-derived reporter ion interference in yeast peptide measurements.

We conducted a two-proteome multiplex (TMTpro 16plex) proteomics experiment, following a similar approach to previous studies (8, 9, 10, 11, 13, 14, 15, 16, 25, 26, 27, 28, 29, 30). The experiment involved proteomes from human Jurkat E6-1 cells and the budding yeast strain W303-1A (Fig. 1*A*). Human peptides constituted the majority of each sample and served as a stable quantitative background. Yeast peptides of comparatively low abundance were varied in defined ratios across four groups of three technical replicates each, allowing for fold change calculation and differential expression testing. Three reporter ion channels (128N, 129N, and 130N) were completely free of yeast peptides, thereby providing a direct quantitative readout of human-derived reporter ion interference at the MS2 level. This setup allowed us to explore and predict reporter ion interference at the MS2 level using multiple linear regression modeling, as further explained in the subsequent sections. An additional sample without peptides was prepared in parallel using TMT label 126C to interrogate the effect of hydrolyzed and quenched label on ion interference (Fig. 1*A*).

### Cell Culture

*Saccharomyces cerevisiae* yeast cells W303-1A were cultured shaking (200 rpm) at 30 °C until mid-log phase (OD_600_ nm ∼1) in 50 ml rich medium (YPD; 1% yeast extract, 2% peptone, 2% glucose). Cells were harvested by filtration (Protran 0.45 μm nitrocellulose membrane) and frozen in liquid nitrogen. Human Jurkat E6-1 cells were cultured in Roswell Park Memorial Institute 1640 media (Gibco), supplemented with 10% fetal bovine serum (Gibco), penicillin, and streptomycin (Gibco). Cells were pelleted and washed with 1 × PBS prior to freezing in liquid nitrogen.

### Proteomic Sample Preparation

Frozen yeast cell pellets were resuspended in lysis buffer (8 M urea, 50 mM Tris buffer pH 8.0, 150 mM NaCl, 1 mM PMSF, 5 mM sodium butyrate, benzonase, and protease inhibitor cocktail) and subsequently lysed by bead-beating (FastPrep-24 device, MP Biomedicals). Frozen Jurkat E6-1 cells were dissolved in lysis buffer and lysed *via* sonication. After removal of insoluble debris by centrifugation at 16,000*g* at 4 °C for 10 min, yeast and human proteins were precipitated by the addition of three times the volume of cold acetone and subsequently incubated overnight at −20 °C. Proteins were pelleted by centrifugation at 15,000*g*, dissolved in 8 M urea, 50 mM ammonium bicarbonate buffer, and treated with 10 mM DTT for 45 min to reduce protein disulfide bonds. Alkylation of reduced cysteines was performed by adding iodoacetamide to a final concentration of 20 mM, followed by incubation for 30 min in the dark. The remaining iodoacetamide was quenched, and samples were diluted to 4 M urea for digestion with Lys-C 1:50 (w/w). After incubation for 2 h at room temperature, samples were further diluted to a final concentration of 1 M urea, and digestion with trypsin 1:50 (w/w) was carried out overnight at 37 °C. Finally, tryptic peptides were desalted using SepPak C18 cartridges (Waters), and eluates were shortly vacuum centrifuged and subsequently lyophilized.

### Two-Proteome Controlled Experiment

Lyophilized tryptic yeast and human peptides were reconstituted in 0.1% TFA to reach a peptide concentration of 3 μg/μl each. Four distinct artificial two-proteome mixtures were created as follows: A constant amount of human peptide solution (300 μl) was initially prepared for each mixture. Then, a comparatively small, variable amount of yeast peptide solution was added to each solution, namely 0, 18, 27, and 36 μl, respectively, resulting in theoretical fold changes of 1.33, 1.5, and 2.0. The yeast-human mixtures were subsequently lyophilized and again reconstituted in 120 μl 100 mM TEAB (Sigma) each. Next, each mixture was split into three technical replicates, resulting in 12 peptide samples in total. TMT labeling was conducted according to a labeling scheme that minimizes isotopic interference across groups (Fig. 1*A*). Additionally, a thirteenth sample without peptides was labeled in parallel using TMT label 126C.

### TMT Labeling

Dried TMTpro 16plex 0.5 mg label reagents (Thermo Fisher Scientific) were resuspended in 30 μl anhydrous acetonitrile (ACN) and mixed with 30 μl 100 mM TEAB peptide solution, resulting in a ratio of TMT label to peptide of approximately 2:1 (w/w) and a final peptide concentration of about 4.2 μg/μl per 60 μl labeling reaction. After incubation for 1 h, a small aliquot of each reaction was combined in 100 μl of 0.1% TFA. This pooled sample was measured on the mass spectrometer to ascertain a labeling efficiency of >99% in each channel by using an in-house R script (https://github.com/maxperutzlabs-ms/Publication_Resources) for the analysis. The labeling reactions were subsequently quenched by the addition of 7 μl of 5% hydroxylamine and incubation for 25 min at 400 rpm at RT. Finally, all 13 labeled samples were combined at 1:1, desalted *via* SepPak C18 cartridges, vacuum centrifuged, and lyophilized prior to neutral pH fractionation.

### Neutral pH Fractionation

Dried labeled peptides were reconstituted in 100 μl 10 mM ammonium formate pH 6.8. After sonication, the solution was injected into UltiMate 3000 Dual LC pHPLC System equipped with a C18 column (xBridge Peptide BEH C18, 25 cm x 4.6 mm, 3.5 μm, Waters). Fractions of 1 ml were collected during separation on a 5 to 50% ACN 1 ml/min gradient in 10 mM ammonium formate buffer pH 6.8 at a flow rate of 1 ml/min. The resulting 1 ml fractions were vacuum centrifuged to evaporate the ACN and then pooled according to a cyclical pooling scheme (31) to create six sample pools of reduced complexity (labeled P1-P6). The pools were desalted *via* SepPak C18 cartridges and stored at −80 °C until measurement on the LC-MS system.

### Acetylated Peptide Enrichment

Acetylomes were purified using the PTMScan Acetyl-Lysine Motif Kit (Cell Signaling). Six hundred micrograms of peptides were dissolved in 175 μl of 1 × PTMScan IAP buffer. An appropriate amount of antibody bead slurry was washed four times with PBS, added to the peptide solution, and incubated for 2 hours at 4 °C with end-over-end rotation. The beads were subsequently washed twice in 1 × IAP and 3 × with HPLC-grade water (Fisher Chemical). Peptides were eluted with 2 x 20 μl of 0.15% TFA. Eluates were united and desalted using C18 StageTips (32). The enrichment protocol resulted in a relative lysine-acetylated peptide abundance of 39.2%.

### Phosphorylated Peptide Enrichment Using TiO_2_

An aliquot (peptide: TiO_2_ resin = 1: 6) of TiO_2_ (Titansphere TiO, GL Sciences, 5020-75000) was washed twice with 50% methanol (Fisher Chemical, A456-212) and twice with glycolic acid solution (1 M glycolic acid (Sigma Aldrich, 124737-25G), 70% ACN (VWR, 83639.320), 3% TFA (Thermo Fisher Scientific, 28903)). Peptides were dissolved in glycolic acid solution, mixed with the TiO_2_ resin, incubated rotating at room temperature for 30 min, transferred onto Mobicol columns (MoBiTec, M1003, M2110), and shortly centrifuged in a table centrifuge to remove unphosphorylated peptides. The resin was washed twice with glycolic acid solution, twice with 200 μl 70%, ACN 3%, TFA, and twice with 1% ACN, 0.1% TFA. Phosphorylated peptides were eluted twice using 150 μl 300 mM ammonium hydroxide (VWR, 1.05432.1000); eluates were united and immediately acidified with conc. TFA to a pH of 2.5. Samples were desalted using a standard C18 StageTip protocol (32). The enrichment protocol resulted in a relative phosphorylated peptide abundance of 95.3%.

### Measurement on the LC-MS System

Unless specified otherwise, the results are based on data generated from the yeast-human fraction pools of reduced sample complexity (labeled P1-P6) measured *via* MS2-based quantification. In brief, for each of the six pools, a total of 200 ng peptide material was measured on a Q Exactive HF-X Orbitrap mass spectrometer (Thermo Fisher Scientific), coupled to the LC-system with a nano-spray ion-source using coated emitter tips (PepSep, MSWil). Peptides were separated along a 2h segmented linear gradient of 2-10-30-40% solvent B (80% acetonitrile, 0.08% formic acid; solvent A 0.1% formic acid) on an Ultimate 3000 RSLC nano-flow chromatography system (Thermo Fisher Scientific) using a pre-column for sample loading (Acclaim PepMap C18, 2 cm × 0.1 mm, 5 μm, Thermo Fisher Scientific), and a C18 analytical column (Acclaim PepMap C18, 50 cm × 0.75 mm, 2 μm, Thermo Fisher Scientific) for separation, at a flow rate of 230 nl/min. On the mass spectrometer, the following instrument settings were applied. For MS1 scans: resolution 120 k, target automatic gain control (AGC) 1e6, maximum IT 60 ms; for MS2 scans: resolution 45 k, target AGC 2e5, maximum IT 120 ms, respectively. Precursor ions were targeted for fragmentation in a top 20 method using a dynamic exclusion time of 20 s. Target ions were isolated at an isolation window width of 0.7 Th and fragmented at normalized collision energy of 35. Precursor ion charge states of 1 or >7 were excluded.

**Fig. 2:**
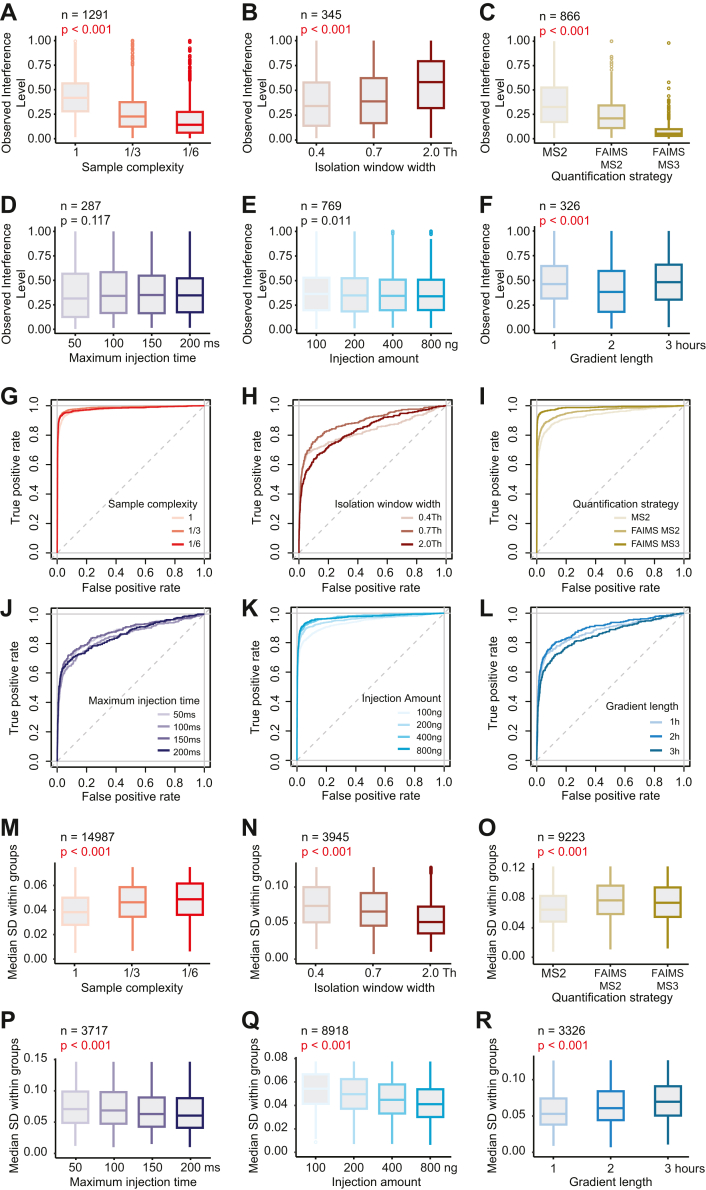
**Dependence of ion interference and differential expression analysis on measurement parameters.***A*–*F*, distributions of the observed interference level (OIL) of yeast peptide features independently quantified in all conditions within a comparison. *p*-values denote statistical significance for overall differences between conditions (Friedman test). The examined measurement parameters are as follows: *A,* Varying sample complexities. Numbers 1, 1/3, and 1/6 correspond to factors of sample complexity reduction *via* fractionation—*e.g.* 1 implies just one measurement run of the unfractionated sample, while 1/6 implies six measurement runs in total of the six fractionated parts with reduced sample complexity. *B*, varying isolation window widths, in Thomson (Th). *C*, varying quantification strategies. FAIMS-MS3 measurements were also real time-searched (RTS). *D*, varying maximum injection times, in milliseconds (ms). *E*, Varying injection amounts, in nanogram (ng). *F*, varying gradient lengths, in hours (h). *G*–*L*, ROC curves calculated from yeast and human peptide features independently quantified in all conditions within comparisons. Measurements are the same as in (*A*–*F*). The true positive rate was calculated as the fraction of yeast peptide features correctly classified as differentially expressed at a certain significance level. The false positive rate was calculated as the fraction of human peptide features incorrectly classified as differentially expressed at a certain significance level. *M*–*R*, distributions of the median within-group standard deviation of yeast and human peptide features independently quantified in all conditions within a comparison. Measurements are the same as in (*A*–*F*). *p*-values denote statistical significance for overall differences between conditions (Friedman test). FAIMS, high-field asymmetric-waveform ion mobility MS.

Measurements for the interrogation of varying measurement-specific parameters (Fig. 2) were performed on Q-Exactive HF-X (Thermo Fisher Scientific) mass spectrometers with generally similar instrument settings—with one exception: For the comparison of varying quantifications strategies, all measurements were performed on an Orbitrap Eclipse Tribid mass spectrometer (Thermo Fisher Scientific), as described in detail in supplemental Table S1. The instrument settings were selected to optimize the performance of each individual quantification method while ensuring maximum comparability.

Acetyl (K) and phosphopeptide enriched samples were measured as follows: LC-MS/MS analysis was performed on an UltiMate 3000 RSLC nano-HPLC System (Thermo Fisher Scientific), containing both a trapping column for peptide concentration (PepMap C18, 5 × 0.3 mm, 5 μm particle size) and an analytical column (PepMap C18, 500 × 0.075 mm, 2 μm particle size (Thermo Fisher Scientific)), coupled to a Q Exactive HF-X Orbitrap (with HCD, higher-energy collisional dissociation mode) mass spectrometer *via* a Proxeon nanospray flex ion source (all Thermo Fisher Scientific). Peptides were separated along a 2 h segmented linear gradient of 2-10-30-40% solvent B (80% acetonitrile, 0.08% formic acid; solvent A 0.1% formic acid) at a flow rate of 230 nl/min. For acquisition of MS2 spectra, the instrument was operated in a data-dependent mode with dynamic exclusion enabled. The scan sequence began with an Orbitrap MS1 spectrum with the following parameters: resolution 120 k, scan range 375 to 1400 m/z, target AGC 3e6, and maximum IT 60 ms. The top 15 precursors were selected for MS2 analysis (HCD) with the following parameters: resolution 45 k, AGC 2e5, maximum IT 200 ms, isolation window 0.7 m/z, scan range 350 to 1500 m/z, and normalized collision energy 35. The minimum AGC target was set at 8 × 10^3^, which corresponds to a 4 × 10^4^ intensity threshold. Peptide match was set to preferred. In addition, unassigned, singly, and >6+ charged species and isotopes were excluded from MS2 analysis, and dynamic exclusion was set to 30 s.

### Database Search

Raw files were searched with MaxQuant (33) (version 1.6.14) against a concatenated yeast and human database (Uniprot release 2020.01, canonical sequences) and a common contaminants database while allowing up to two missed cleavages on tryptic peptides. Search parameters considered oxidation of methionine as well as N-terminal acetylation as variable modifications and included carbamidomethylation of cysteine residues as a fixed modification. TMTpro label modifications were allowed on both N-termini and lysine residues (as variable or fixed modifications, depending on the analysis requirements). Search settings additionally considered variable phospho (STY) or acetyl (K) peptide modifications for raw files of acetyl (K) and phospho (STY)-enriched samples. Raw files generated by FAIMS quantification were split according to scan-specific compensation voltages using Freestyle (Thermo Fisher Scientific). The resulting files were then treated as any other raw file for subsequent database searching. All search results were filtered to reach an expected false discovery rate (FDR) of 1% at the PSM level.

Some data were generated using alternative search tools (supplemental Table S2). *FragPipe*: Thermo raw files were converted to mzML using msConvert (34) and searched (closed search) against a concatenated yeast and human database including common contaminants using FragPipe (35) (version 16.0). Carbamidomethylation on cysteines was specified as a fixed; oxidation (methionine), N-terminal acetylation, and TMTpro 16plex label were searched as variable. PSM validation was conducted with Percolator (36), PSMs were filtered at 1% FDR, and protein inference was conducted with ProteinProphet (37) filtered at 1% FDR. Additional filters using TMT Integrator: minimum PSM probability: 0.9, minimum best peptide probability: 0.9, normalization: “None”. *Proteome Discoverer*: Raw files were searched against a concatenated yeast/human database using Proteome Discoverer MS Amanda (38) v2.0. Search parameters: Carbamidomethylation on cysteines was set as static, N-terminal TMTpro 16plex modification as variable modification. Oxidation (methionine), deamidation (asparagine and glutamate), and TMTpro 16plex label were set as variable. Maximum number of missed cleavages: 2. Results were filtered at 1% FDR on PSM and protein level using Percolator (36) and at a minimum MS Amanda Score of 150 at PSM level.

### Raw File Data Extraction

In addition to database searching, select spectral features like m/z, intensity, and noise values of centroided peaks were extracted directly from Thermo raw files *via* an in-house command line application https://github.com/fstanek/rawStallion that uses the Thermo RawFileReader library. The combined output was imported into the R statistical environment (39) and further analyzed there.

### Reporter Intensity Inference and Isotopic Impurity Correction

Reporter ion intensities were inferred by taking the maximum observed peak intensity value within a ± 0.002 Th window at the expected reporter ion m/z. For this task, we adapted code from the R proteomics software MSnbase (40) and utilized the reporter ion classes pre-defined therein. Further, we used matrix algebra to correct for TMT isotopic impurities as implemented in MSnbase (40). The impurity matrix was calculated manually from the product sheet provided by the manufacturer (lot number VC294906).

### Estimation of Run-Specific Peptide Density

Retention time and m/z values of all identified, uniquely charged peptide features were used to construct a run-specific two-dimensional kernel density estimate, evaluated on a 200 × 200 grid. The calculation employed the kde2d function from the R package MASS (41). Resulting density values were rescaled to sum up to 1. Further, the square root of all density values was taken to shrink extreme differences in the m/z and retention time plane, and density values of 0 were substituted with the minimum calculated positive density. Finally, the 200 × 200 density grid was interpolated for each individual PSM at the precursor-specific m/z and retention time coordinates using the interp.surface function from the R package fields (42).

### Calculation of Variables

#### OIL

The “observed interference level” (OIL) denotes the PSM-specific fraction of interference-induced reporter ion signal at MS2 level. It is calculated as the ratio of two intensity averages derived from the same MS2 scan: In the numerator, the average normalized reporter ion intensity of channels in which the quantitative signal can exclusively be ascribed to interference-derived signal stemming from a uniform background; in the denominator, the average normalized reporter ion intensity of all other channels that are affected by the same uniform background interference. To ensure values between 0 and 1, calculated OIL values above 1 are capped at 1. For yeast PSMs in the yeast-human mixture experiment, the formula is thus given by

(1) $$OIL=\min\;\left\{\frac{\text{Avg}\;\left(I_{128N\;},I_{129N},I_{130N}\right)}{\text{Avg}\;\left(I_{128C\;},I_{129C},I_{130C},I_{131C\;},I_{132C},I_{133C},I_{132N\;},I_{133N},I_{134N}\right)},1\right\}$$

where $I_{c},c\in \left\{126C,127N,\ldots ,134N\right\}$ is the normalized reporter ion intensity in channel c.

#### PPF

The “precursor purity fraction” (PPF) denotes the PSM-specific fraction of signal originating from precursor peptide ions (including isotopes) with respect to the total measured ion signal in the isolation window of bordering MS1 scans. It is similar to existing ion purity metrics like "precursor ion fraction" (PIF) (43). First, all centroided peak intensities $I_{ip},i\in \left\{-1,+1\right\},p\in \left\{1,2,\ldots ,k_{i}\right\}$ found in the isolation windows of the two bordering MS1 scans indexed by i,i∈{−1,+1} are extracted, where $k_{i}$ denotes the total number of distinct peaks in the isolation window of MS1-scan i. It is then checked if the precursor ion peak can be found among the peaks in the preceding MS1 scan (i=−1) within a margin of error of ± 0.0025 Th. If the check is unsuccessful, the information of the second last MS1 scan is used instead, provided that the precursor ion peak is found there (this was typically the case). We found this situation to occur more frequently for measurements on Exploris and Eclipse instruments than on HF-X Orbitrap instruments. If the precursor ion peak is still not found, its intensity is imputed at the declared precursor m/z by taking the minimum intensity value over all observed peaks in the preceding MS1 scan (i=−1). Concerning the following MS1 scan (i=+1), if the respective isolation window is entirely empty, the scan is skipped and not used in the calculation. Else, if the isolation window is non-empty but the precursor ion peak is not found at the declared precursor m/z (± 0.0025 Th), the precursor peak intensity is again imputed as just described. In the rare event that the precursor peak cannot be found in either of the bordering MS1 scans, the following MS1 scan (i=+1) is skipped. Next, the extracted peak intensities $I_{ip},i\in \left\{-1,+1\right\},p\in \left\{1,2,\ldots ,k_{i}\right\}$ in the isolation windows of bordering MS1 scans are inversely weighted by absolute differences between MS1 retention times $t_{i}$ and the retention time of the MS2-scan $t_{MS2\;}$ that produced the PSM. This results in weighted intensities $I_{ip}^{*}$:

(2) $$d_{i}=\left|{\;t}_{i}-\;t_{MS2\;}\right|,i\in \left\{-1,+1\right\}$$

(3) $$w_{i}=1-\frac{\;d_{i}\;}{\sum_{i}d_{i}\;},i\in \left\{-1,+1\right\}$$

(4) $$I_{ip\;}^{*}=w_{i}{\cdot I}_{ip\;},i\in \left\{-1,+1\right\},p\in \left\{1,2,\ldots ,k_{i}\right\}$$

Next, heavy (+1 Da) and light (−1 Da) isotope peaks of the precursor peptide are determined using a mass error tolerance of ± 0.00125 Th. This results in the subset of peaks $I_{precursor\;}^{*}$ comprising all weighted peak intensities $I_{ip\;}^{*}$ that can be ascribed to the precursor peptide, including heavy and light isotope ions, detected in the isolation window of bordering MS1 scans. The precursor purity fraction PPF is then defined as

(5) $$PPF=\frac{\sum_{i}\sum_{p\;}I_{ip\;}^{*}\cdot 1\left(I_{ip}^{*}\right)}{\sum_{i}\sum_{p}I_{ip\;}^{*}},1\left(I_{ip}^{*}\right)=\{\begin{array}{c} 1\;\text{if}\;I_{ip\;}^{*}\in I_{\text{precursor}\;}^{*}\\ 0\;\text{if}\;I_{ip\;}^{*}\notin I_{\text{precursor}\;}^{*} \end{array},\;i\in \left\{-1,+1\right\},\;p\in \left\{1,2,\ldots ,k_{i}\right\}$$

#### TIW

The “total intensity in the isolation window” (TIW) is defined as

(6) $$TIW=\sum_{i}\sum_{p}I_{ip\;}^{*},i\in \left\{-1,+1\right\},p\in \left\{1,2,\ldots ,k_{i}\right\}$$

This equation uses the same notation as the PPF calculation (Equation 5), where TIW is the denominator.

#### PIC

The “total peptide ion current” (PIC) is calculated as the total ion current of an MS2 scan minus the total reporter ion intensity of the same scan. This metric therefore equals the sum of all peak intensities stemming from peptide (fragment) ions in an MS2 scan, including the unfragmented precursor ion if not fully fragmented.

### Multiple Linear Regression Modeling

We employed multiple linear regression modeling to model and predict the interference-induced reporter ion signal in the yeast-human mixture experiment. The model formula is given by

(7) Y∼0+nonprecursor:rawfileCharge+noiseEstimate

where

Y is the dependent variable of the model and denotes the total reporter ion intensity of channels 128N, 129N, and 130N of yeast PSMs (*i.e.* the observable human-derived reporter ion interference).

nonprecursor=TIW⋅(1−PPF). This predictor variable denotes the total intensity of non-precursor interfering ions observed in the isolation window.

rawfileCharge is a categorical variable describing the charge state of the precursor ion as reported by the mass spectrometer and documented in the raw file. Charge states above 3 were set to 3 to ensure sufficient numbers of observations in each category.

noiseEstimate is a PSM-wise estimate of the signal of non-observable interfering ions in the isolation window. It is calculated as the product of the average reported noise value of peaks in the isolation window of the preceding MS1 scan and the interpolated run-specific peptide density at the precursor-specific m/z and retention time coordinate.

Further, “0” denotes an intercept fixed at 0, and the colon denotes interaction effects between predictor variables. The model parameter coefficients are estimated *via* robust linear regression modeling with bisquare weighting using the rlm function from the R package MASS (41). This robust estimation procedure accounts for outliers as well as extreme heteroscedasticity. Data from each unique raw file is modeled with its own distinct set of parameters.

Moreover, we propose an extended model that comprehensively explains the total reporter ion intensity of MS2 scans in any MS2-quantified multiplex proteomics experiment. The model formula is given by

(8) Y∼0+precursor:pepClass+nonprecursor:rawfileCharge+noiseEstimate

where

Y is the dependent variable of the model and denotes the total reporter ion intensity in MS2 scans.

precursor=PIC⋅PPF

pepClass is a categorial variable that specifies empirical classes of specific combinations of observed precursor peptide characteristics. It is calculated using a greedy classification algorithm that continuously creates binary splits in peptide characteristic predictor variables in order to best explain observed differences in the fragmentation efficiency of precursor peptides.

nonprecursor=PIC⋅(1−PPF)

rawfileCharge and noiseEstimate are defined as above.

As in the previous model, parameter estimates are calculated *via* robust linear regression modeling with bisquare weighting. Data from each unique raw file are modeled with its own distinct set of parameters. Further, separate modeling is performed for each unique compensation voltage in data recorded *via* FAIMS-MS2.

### Calculation of the Estimated Interference Level

Fitting a linear regression model (Equation 8) to MS2-quantified multiplex proteomics data results in estimated model parameters $\hat{\beta }$ that can be used to predict PSM-wise levels of reporter ion interference in MS2 scans. We define the “estimated interference level” or EIL in short as

(9) $$EIL=\min\left\{1-\frac{x_{precursor}^{T}{\hat{\beta }}_{precursor}}{\;x^{T}\hat{\beta }},1\right\}$$

where

$\hat{\beta }={\left({\hat{\beta }}_{1},{\hat{\beta }}_{2},\ldots ,{\hat{\beta }}_{k}\right)\;}^{T}$ is the k x 1 vector of estimated parameter coefficients, x is a k x 1 vector containing the PSM-specific realizations of predictor variables, $x^{T}\hat{\beta }$ is therefore the fitted value of the model, that is, the PSM-specific model estimate of total reporter ion intensity.

${\hat{\beta }}_{precursor}={\left({\hat{\beta }}_{1},{\hat{\beta }}_{2},\ldots ,{\hat{\beta }}_{p}\right)\;}^{T}$ is a p x 1, p < k subset of $\hat{\beta }$ containing only the coefficients that describe the contribution of the precursor peptide to Y,

$x_{precursor\;}$ is the corresponding p x 1, p < k subset of x, such that

$x_{precursor\;}^{T}{\hat{\beta }}_{precursor}$ is the PSM-specific model estimate of reporter ion intensity generated only by precursor peptide ions, including heavy and light isotopes.

### Interference Correction

Similar to Savitski *et al.* (23), we propose a PSM-wise interference correction algorithm that generates interference-corrected reporter ion intensities from the estimated interference at MS2 level. The major difference to the original approach lies in using a different kind of impurity metric for the calculation—the EIL (Equation 9)—instead of an impurity metric that is derived from the MS1 isolation window purity like 1-PPF. Additionally, our algorithm replaces interference-corrected intensity values that fall below a certain threshold with a spectrum-wise minimum. The calculation for the yeast-human mixture experiment is given by

(10) $$\bar{I}=\frac{1\;}{\left|C\right|\;}\sum_{c}\;I_{c},c\in C=\left\{128C,129N,\ldots ,134N\right\}$$

where $I_{c}$ denotes the untransformed, normalized reporter ion intensity in reporter channel c; $\bar{I}$ thus equals the average normalized reporter ion intensity. Importantly, missing values as well as empty channels are excluded in the calculation. Then, corrected normalized reporter ion intensities $I_{c}^{corrected}$ are calculated as

(11) $$I_{c}^{corrected}=I_{c}-\;\bar{I}\cdot \min\left\{\;\text{EIL},0.8\;\right\}$$

In this formula, EIL values are capped at an arbitrary cutoff of 0.8 to mitigate overcorrection. Finally, corrected reporter intensities $I_{c}^{corrected}$ that fall below the minimum of observed MS2 peak intensities are replaced with that minimum to mitigate potential artefacts due to overcorrection. Missing reporter intensity values are substituted equally.

### Site-To-Protein Normalization

Utilizing PSM-wise EILs (Equation 9), we devised a simple algorithm to normalize PTM site intensities to the corresponding protein levels and account for differences in reporter ion interference. Some aggregation steps were necessary beforehand: PSM-wise reporter intensities of unmodified peptides and their corresponding EIL values were aggregated to protein level using the information contained in MaxQuant’s protein table (supplemental Table S3). In more detail, for all PSMs belonging to a single protein group, normalized PSM level reporter intensity values were combined by summation, and PSM-wise EIL values were aggregated to a single EIL value by calculating the corresponding (intensity-weighted) averages. Analogously, PSM level information of modified peptides was aggregated to PTM site level using the information contained in MaxQuant’s acetyl (supplemental Table S4) and phospho-site tables (supplemental Table S5). If quantified *via* FAIMS-MS3, feature-wise EIL values were set to 0.

The site-to-protein normalization algorithm can be formulated as follows: Consider that a PTM site s matches to exactly one quantified protein group p. First, the difference in EIL values is calculated as

(12) $$\Delta EIL={EIL}_{s}-\;{EIL}_{p}$$

where ${EIL}_{s}$ and ${EIL}_{p}$ denote the estimated interference levels of site s and protein p, respectively. Assume that ΔEIL>0, meaning that the estimated interference level is smaller on protein than on PTM site level. Consequently, the algorithm will only adjust the reporter ion intensities of protein p by the addition of uniform interference background to reach equality in interference levels. The interference-adjusted protein reporter intensities are calculated as

(13) $$I_{p,c}^{adj}=I_{p,c}+\Delta_{Interference},$$

where $I_{p,c}$ is the reporter intensity in channel c of protein p, and $\Delta_{Interference}$ is the uniform interference background that needs to be added to reach equal interference levels; $I_{p,c}^{adj}$ therefore is the interference-adjusted reporter intensity in channel c of protein p. $\Delta_{Interference}$ is obtained by

(14) $$\Delta_{Interference}={\bar{I}}_{p}\cdot \frac{1-{EIL}_{p}}{1-{EIL}_{s}}-{\bar{I}}_{p},\text{where}$$

(15) $${\bar{I}}_{p}=\frac{1\;}{\left|C\right|\;}\sum_{c}\;I_{p,c},c\in C$$

In the last equation, C denotes the set of all non-empty channels used in the experiment; ${\bar{I}}_{p}$ thus equals the average normalized reporter ion intensity of protein p. Finally, site-to-protein ratios between reporter intensities $I_{s,c}$ of PTM site s and interference-adjusted reporter intensities ${\;I}_{p,c}^{adj}$ of protein p are calculated for each channel c as

(16) $$r_{s,p,c}=\frac{I_{s,c}}{I_{p,c}^{adj}}$$

Since the absolute values of $r_{s,p,c}$ have no meaning (as they are only to be compared between channels), they are subsequently normalized using the median value of all ratios,

(17) $$r_{s,p.c}^{norm}=\frac{r_{s,p,c}}{{Median}_{c\in C}\left(r_{s,p,c}\right)}$$

such that a median ratio of 1 is reached.

Analogously, if ΔEIL<0, instead the PTM site intensities of site s are interference-adjusted to reach equal interference levels prior to calculating the site-to-protein ratios.

### Data Transformation, Normalization, and General Analysis

PSM-wise reporter ion intensities were log_2_-transformed and subsequently normalized using a cyclic LOESS normalization strategy contained in the R package limma (44) or by using DESeq2’s size factors (45) for normalization as described previously (46). After normalization, reporter ion interference was assumed to affect all channels uniformly. Notably, for many of the calculations described above, the log_2_-transformed normalized reporter ion intensities were transformed back from log-space by applying the corresponding inverse function on the transformed data (*i.e.* exponentiation with base 2). Statistical testing for differential expression between groups was conducted *via* the Limma-trend testing procedure (47) on log_2_-transformed normalized reporter ion intensities. Various additional R packages were used in the analysis and data visualization (https://cran.r-project.org/web/packages/plot3D/index.html) (48, 49, 50).

## Results

### Experimental Design

To interrogate ion interference, we designed an artificial two-proteome multiplex proteomics experiment comprising TMTpro 16plex-labeled tryptic peptides of budding yeast (W303-1A) and human cells (Jurkat E6-1) (Fig. 1*A*). While human peptide abundance remained constant throughout all samples, yeast peptide abundance was varied in defined ratios across four sample groups of three technical replicates each. Importantly, the mass ratio of human to yeast peptides was set to approximately 10:1 (Fig. 1*A*) in order to minimize any interference from yeast while still ensuring a sufficiently large number of quantified yeast peptides to support the analysis. Similar to the triple knockout (TKO) proteomic standards (51, 52, 53), this system allowed direct assessment of the degree of reporter ion interference at MS2 level *via* the three reporter channels 128N, 129N, and 130N in quantified yeast peptides (Fig. 1*B*). The profile of total measured yeast and human reporter intensities supports this premise (Fig. 1*C*) by illustrating how human signal interfered in yeast peptide measurements. Moreover, to determine the contribution of chemical label background to ion interference, one sample of excessive TMT (126C) label was used without peptides. However, the corresponding signal was negligible in comparison to the interference-induced reporter ion signal in channels 128N, 129N, and 130N (Fig. 1*C*). This implicates that ion interference is generally not caused by quenched or hydrolyzed TMT label molecules but instead by labeled peptide ions that are co-isolated and cofragmented during MS2-based quantification of target peptides.

### Measuring Interference at MS2 Level

Using this direct quantitative readout on interference at MS2 level, we first explored how commonly varying sample and measurement parameters affect interference. In particular, we were interested in the factors which, all other things being constant, altered the extent of reporter ion interference. This led us to investigate the effect of alterations in six distinct measurement parameters, changed one at a time, while everything else was held equal. The examined parameters comprised the sample complexity (represented by varying degrees of sample fractionation), isolation window width, quantification method (MS2, FAIMS-MS2, and FAIMS-MS3 RTS (real-time search)), maximum MS2 injection time, sample injection amount, and HPLC gradient length. Each parameter was examined independently, dividing the whole examination into six separate experiments. To always focus on a single parameter change in isolation, we conducted the data analysis in a specific manner (supplemental Fig. S1): First, for all conditions to be compared in an experiment, we determined all quantified peptide features with distinct combinations of amino acid sequence, chemical modifications, and charge state. Second, these unique peptide features were filtered for independent quantification in all conditions to be compared. Consequently, the same peptide features across different conditions were always compared to each other in the subsequent statistical analyses (supplemental Fig. S1). This guaranteed comparability by preventing bias from varying depths of quantification which were directly linked to alterations in some of the six parameters (such as changes in sample complexity and gradient length). Further, this strategy removed any dependence on the stochasticity inherent to data-dependent acquisition.

For each yeast peptide feature, we then calculated the average fraction of interference-induced reporter ion intensity (*i.e.* intensity in the yeast-free channels 128N, 129N, 130N) with respect to the reporter ion intensity across all other channels. This fraction was termed the “observed interference level” or OIL in short (Equation 1). It would assume 0 in the absence of any measurable reporter ion interference in channels 128N, 129N, and 130N and could reach up to 1, at which point the entire quantitative signal in a yeast scan was attributed to human-derived interference. Our findings support previous literature results (7, 8, 9, 10, 54) by showing that reducing sample complexity (Fig. 2*A*) or isolation window widths (Fig. 2*B*) effectively alleviate interference at the MS2 level. We also observed a previously described reduction in interference for FAIMS-MS2 quantification over standard MS2 quantification (13) and almost completely interference-free TMT quantification *via* FAIMS-MS3 RTS (10) (Fig. 2*C*). Varying maximum MS2 injection times (Fig. 2*D*), as well as varying sample injection amounts (Fig. 2*E*) yielded no remarkable change in reporter ion interference. However, our data show lowest levels of interference for intermediate gradient lengths (Fig. 2*F*). We attribute this finding to altered peptide elution profiles, which—combined with the applied instrument settings—result in the fragmentation of peptides at more favorable elution times (55). Notably, all the above-mentioned changes in interference are equally reflected by an accompanied degree of ratio compression in calculated yeast peptide fold changes (supplemental Fig. S2, *A*–*F*).

Next, we investigated how these changes in ion interference impact differential expression (DE) analysis. This is of particular interest since ratio compression evidently reduces the effect size of group differences (supplemental Fig. S2, *A*–*F*), which in turn negatively impacts statistical power. Reporter intensity values were first log_2_-transformed to ensure normality of errors. We then conducted pairwise differential expression testing *via* the Limma-trend testing procedure (47) between the two sample groups 100:9 (channels 131C, 132C, 133C) and 100:6 (channels 128C, 129C, 130C), resulting in the theoretical fold changes of 1.0 for human and 1.5 for yeast peptides, respectively (Fig. 1*A*). Receiver operating characteristic (ROC) curves were calculated to compare the diagnostic abilities of correctly classifying yeast peptide features as differentially expressed *versus* human peptides as nondifferentially expressed (Fig. 2, *G*–*L*). The data revealed that ion interference is not the only factor affecting DE analysis results in isobaric labeling-based quantification, since the varying levels of interference (Fig. 2, *A*–*F*) failed to sufficiently explain ROC curve differences (Fig. 2, *G*–*L*). In addition, the reporter ion signal strength appears pivotal by influencing measurement precision (*i.e.* variance within groups) *via* an observable mean-variance trend (supplemental Fig. S3), which in turn affects statistical power. We observed significant changes in reporter ion signal (supplemental Fig. S2, *G*–*L*) and corresponding within-group variances (Fig. 2, *M*–*R*) for all comparative measurements, which ultimately help to explain differences in ROC curves when paired with measured changes in observed interference levels (Fig. 2, *A*–*F*). For example, despite higher levels of interference in the more complex samples (Fig. 2*A*), the increased precision due to the higher overall signal (Fig. 2*M* and supplemental Fig. S2*G*) compensated for the loss of accuracy in statistical testing (Fig. 2*G*). In other words, we observed that the presence of additional reporter ion signal from interference evoked a tradeoff between accuracy and precision, with both roughly balancing each other out in their combined influence on DE analysis. Importantly, in order to focus on the effect of sample complexity in isolation, we intentionally did not scale up the total injection amount across all samples of reduced complexity compared to the unfractionated sample (supplemental Fig. S1). Incidentally, an increase in the total injection amount showed improved classification results due to a gain in precision from higher overall reporter ion signal (Fig. 2, *K* and *Q*, and supplemental Fig. S2*K*). Hence, performing fractionation combined with an increased total sample load distributed across multiple measurements should benefit DE analysis while at the same time mitigate the effect of ratio compression.

Regarding isolation window widths, our data indicated that the increased precision due to an overall increase in reporter ion signal with larger isolation window widths (Fig. 2*N* and supplemental Fig. S2*H*) can lead to improved classification results (Fig. 2*H*), even if that comes at the cost of higher levels of interference and ratio compression (Fig. 2*B* and supplemental Fig. S2*B*). Notably, in addition to a general higher transmission efficiency, window widths of 0.7 Th also manage to co-isolate +1 and −1 isotopes of triply charged precursor peptides, thus providing a possible explanation as to why a window of 0.7 Th classified markedly better than a window of 0.4 Th (Fig. 2*H*). On the other hand, a window width of 2.0 Th with a median OIL above 0.5 (meaning that for more than 50% of quantified yeast peptides, at least half of the signal was in fact human-derived) appeared to suffer in accuracy to such an extent that the accompanied increase in precision could not compensate for the loss in statistical power due to waning accuracy (Fig. 2, *B*, *H*, and *N*, and supplemental Fig. S2*B*).

Comparisons between ROC curves of varying maximum injection times and gradient lengths were complicated by differing tendencies to repeatedly quantify identical peptide features due to changes in measurement speed and measurement time, respectively. This somewhat hampers the direct comparability and interpretation of calculated ROC curves. Still, the improved classification result from the measurement with an intermediate gradient length is striking (Fig. 2*L*), which emphasizes the importance of reconciling the chromatographic separation with MS instrument settings that determine the quantification of peptides at specific points in the elution profile for maximum accuracy and precision (55).

Finally, given the applied instrument settings (supplemental Table S1), we observed FAIMS-MS2 and in particular FAIMS-MS3 RTS quantification to considerably outperform standard MS2 quantification in correctly classifying differential expression (Fig. 2*I*). Both FAIMS-MS2 and FAIMS-MS3 quantification seemingly benefitted from increased accuracy due to a notable reduction of interference (Fig. 2*C* and supplemental Fig. S2*C*) without losing too much in precision (Fig. 2*O*). Nevertheless, it is important to put these results into a broader context. So far, we focused on unique peptide features that were filtered for independent quantification across all compared conditions within an experiment in order to avoid any selection bias in the analysis (supplemental Fig. S1). However, alterations in the examined measurement parameters often resulted in substantial differences in the number of unique quantified peptides per condition (supplemental Fig. S2, *M*–*R*). Altogether, we find that maximizing identifications, fold change accuracy (*i.e.* minimizing ion interference), and correct classification of differential expression at the same time is not trivial because there is a tradeoff between the three. The choice of measurement strategy is thus best guided by the available instrumentation, as well as the specific aims and constraints of the particular experiment.

### Modeling Interference at MS2 Level

To gain a more causal and mechanistic understanding of reporter ion interference generation, we examined the observed reporter ion interference of yeast peptide scans in more detail. We speculated that the observed interference level (Equation 1) would be best explained by the total ion composition in the precursor peptide’s isolation window range, which gives direct insight into the degree of unwanted co-isolation and subsequent cofragmentation for MS2-based quantification. This reasoning is not novel. For example, the “PIF” (43), implemented in the quantitative proteomics software platform MaxQuant (33), and other similar ion purity metrics (24, 35, 38) estimate the degree of precursor purity by calculating the fraction of ion signal attributed to the precursor peptide with respect to the total ion signal in the isolation window of bordering MS1 scans. Usually, these purity metrics are used to filter out PSMs considered too impure for reliable quantification. Here, we introduce a purity metric termed “PPF” which equally ranges from 0 to 1 by definition (Equations (2), (3), (4), (5)). Calculated PPF values correlate well with existing purity metrics (supplemental Fig. S4, *A*–*C*) but performed best in the modeling approach described below due to subtle differences in calculation (see methods).

**Fig. 3:**
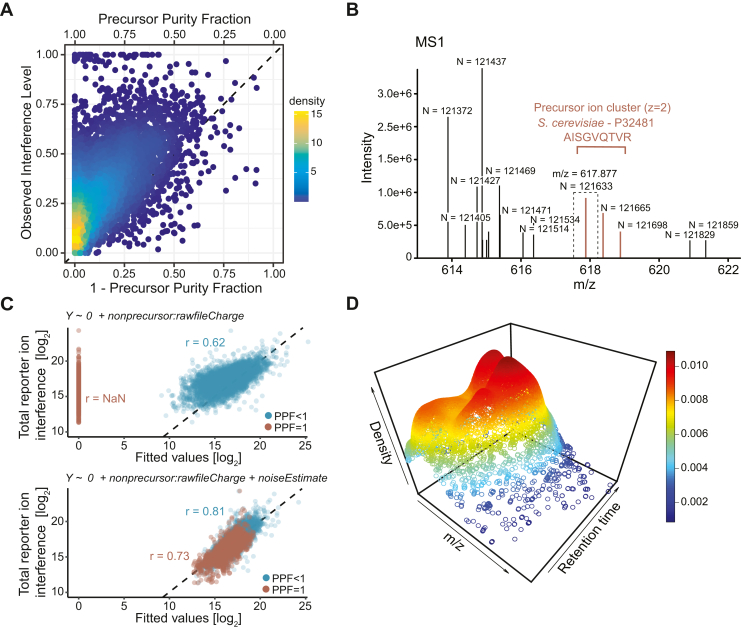
**Modeling reporter ion interference.***A*, relationship between observed interference levels (OIL) and MS1 isolation window impurities (1-PPF). Each data point corresponds to a single yeast PSM. *B*, example of a recorded MS1 spectrum (raw file “20201030_[…]_complexity_P1”, scan number 12583) in which peaks are annotated with their respective noise values N. The colored peak at m/z = 617.877 corresponds to a yeast precursor peptide ion that was subsequently targeted for MS2-based quantification at an isolation window width of 0.7 Th (indicated by *dashed lines*). Despite the absence of visible interference at MS1 level (PPF = 1), the resulting PSM (MS2 scan number 12589) exhibited substantial reporter ion interference at MS2 level (OIL = 0.39). *C*, model prediction of the measured reporter ion interference by two nested linear regression models. Model formulas are depicted on top of each plot. Each data point corresponds to a single yeast PSM. The dashed line represents the identity function (y = x) and therefore reflects a perfect prediction. A pseudo-count of 1 was added to each fitted value before log-transformation in order to ensure finite values. r denotes calculated Pearson correlation coefficients. *D*, 3D-density map showing density estimates calculated for all PSMs in a single measurement run (raw file “20201030_[…]_complexity_P1”). A higher estimated density signifies a larger number of unique peptide features quantified in the vicinity of the m/z and retention time coordinate. PPF, precursor purity fraction.

Remarkably, regardless of which purity metric is considered, the MS1 isolation window purity appears as a rather weak predictor for the actual level of interference observed at MS2 level (Fig. 3*A*). In fact, interference at the MS2 level is substantially underestimated, and even PSMs that appear pure in their respective isolation windows (*i.e.* PPF = 1) exhibited considerable reporter ion interference (supplemental Fig. S5). In agreement with Savitski *et al*. (23), we attribute this discrepancy to how Orbitrap raw files are recorded during measurement. To minimize raw data file size, signals below a certain threshold are considered “noise” and consequently removed. What remains as reduced information in Orbitrap raw spectra are thus a) all peaks with intensities above calculated local noise thresholds that serve as a cutoff and b) noise values specific to each centroided peak (Fig. 3*B*), which are proportional to the aforementioned local noise thresholds†. We assume that by removal of these low-intensity signals, a substantial part of interfering ions remains hidden in the MS1 isolation window range, especially when signal to noise ratios of target precursor ions are low. At the same time, we hypothesize that the noise values contained in the raw data (Fig. 3*B*) could still sufficiently represent the potential strength of the hidden signal.

To explore these hypotheses further, we employed multiple linear regression modeling. The dependent variable of the model (Y) was defined as the spectrum-wise total measured reporter ion interference at MS2 level, calculated as the summed reporter ion intensity in channels 128N, 129N, and 130N of yeast peptide measurements. In line with our previous observation (Fig. 3*A*), a model that only accounts for the observable portion of ion interference in the MS1 isolation window range performed poorly in predicting reporter ion interference (Fig. 3*C*, upper). Next, we adapted the model formula by including a regressor variable that aimed to reflect the potential noise contribution. Assuming that co-isolated interfering ions hidden within the spectral noise are in fact other labeled peptide ions of relatively low abundance, we calculated this additional regressor variable by combining two numerical sources of information *via* multiplication: First, a measure that reflects the potential maximum intensity of such ions, calculated as the average noise value in the precursor peptide’s isolation window range; and second, the estimated frequency of such ions, taken from a measurement-specific empirical peptide density that spans the m/z-retention time plane (Fig. 3*D*). The updated model (Equation 7) managed to accurately predict the interference-induced reporter ion signal for all yeast peptide measurements (Fig. 3*C*, lower), including PSMs that appeared pure in their respective MS1 isolation window range (PPF = 1).

### Estimating Interference at MS2 Level

Accurate estimation of ion interference could prove extremely valuable for the qualitative and quantitative interpretation of MS2-quantified multiplex proteomics experiments. As shown above, a linear regression model fitted to the measured data has the potential to provide this crucial information. However, implementation of such a linear regression model requires direct measurement of reporter ion interference at MS2 level in order to support model training, which is usually not the case in biological experiments.

**Fig. 4:**
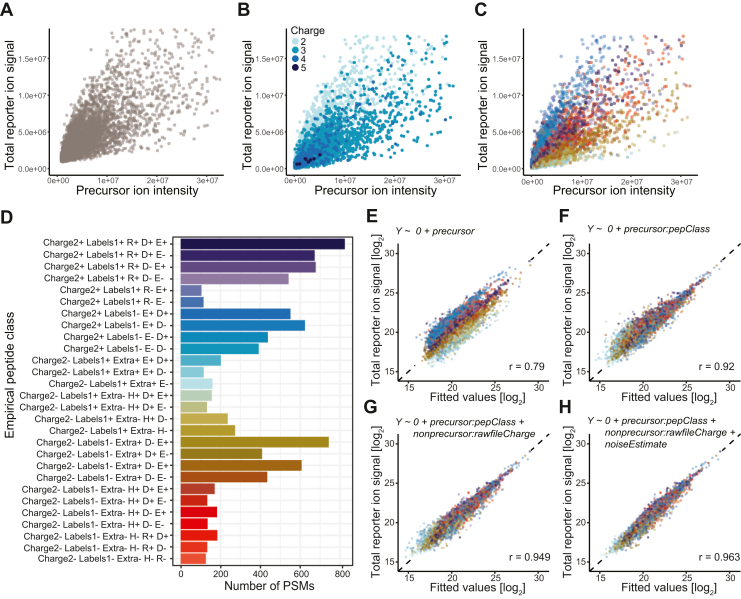
**Modeling total reporter ion signal.***A*, dependence of total reporter ion signal on precursor ion intensity in the MS1 isolation window range. Each data point corresponds to a single yeast or human PSM from raw file “20201030_[…]_complexity_P1”. *B*, the same relationship as in (*A*), colored by precursor ion charge states. *C*, the same relationship as in (*A*), colored by empirical peptide classes of varying fragmentation efficiency. *D*, overview of empirical peptide classes shown in (*C*), as determined by a greedy decision tree-based classification algorithm. Peptide class labels reflect paths in the decision tree from root to leaf (*i.e.* terminal node). Each split (*i.e.* internal node) represents the action of further dividing a peptide class into two distinct sub classes based on a single physicochemical property (*e.g.* contains arginine *versus* contains no arginine, symbolized by “R+” and “R-”, respectively) such that the resulting within-class variance is minimized. The variables available for creating new splits are as follows: the precursor charge state (Charge); the number of TMT-labels (Labels); the presence and absence of amino acids histidine (H), arginine (R), lysine (K), glutamate (E), and aspartate (D); and the existence of an additional positive charge that is not explained by the total number of charged functional groups in the peptide’s chemical structure (Extra). The algorithm ensures a sufficient number of observations within each class (>100) by preventing splits that reduce the number of PSMs below that threshold. *E*–*H*, model prediction of the measured total reporter ion signal by four nested linear regression models. Model formulas are depicted on top of each plot. Each data point corresponds to a single yeast or human PSM from raw file “20201030_[…]_complexity_P1”. The dashed line represents the identity function (y = x) and therefore reflects a perfect prediction. An increasing number of predictor variables were sequentially included into the model from top left to bottom right. Note that differences in slopes will appear as differences in y-intercepts since both axes are log-transformed. r denotes the calculated Pearson correlation coefficient.

We therefore adjusted the model to make it applicable to all MS2-quantified multiplex proteomics data. The dependent variable (Y) was redefined as the total measured reporter ion signal of an MS2 spectrum, thus containing signal stemming from both interference and non-interference (*i.e.* precursor) origin. Naturally, most of this signal is expected to originate from the precursor peptide ion that was targeted for MS2, which makes it essential that the model accurately reflects this contribution to the total signal. However, this task is complicated by a strong divergence in the relationship of precursor and reporter ion intensities, presumably due to peptide-specific differences in the fragmentation efficiencies (Fig. 4*A*). A part of this variation in the fragmentation behavior could be explained by differences in precursor ion charge state (Fig. 4*B*). However, even within individual charge states, a significant level of heterogeneity remained unexplained. Therefore, we tested whether this could be explained by other peptide characteristics. Remarkably, we found that the majority of the observed variance could be attributed to distinct empirical precursor peptide classes derived from the data itself that group peptides based on certain physicochemical properties (Fig. 4, *C* and *D*). Apart from the precursor ion charge state, peptide classes were characterized by the number of TMT labels as well as the presence or absence of specific amino acids. Interestingly, we found that empirical peptide classes also varied greatly in frequency and composition between individual datasets, most likely due to sample and measurement-specific variables (*e.g.* normalized collision energies, chromatography conditions, etc.). Ultimately, we developed a greedy decision tree algorithm that automatically infers a distinct set of empirical peptide classes to optimally group all PSMs in each new dataset encountered (Fig. 4, *C* and *D*). Class variables determined in this way could then be incorporated as separate categorical regressor variables into the linear regression model, which makes the model unique for each new dataset trained on.

Further, we encountered a second hurdle. In most datasets, linear model fitting was hampered by a slight nonlinear relationship between the total MS1 intensity signal in the isolation window (Equation 6) and the total reporter ion signal (supplemental Fig. S6, *A* and *B*, upper rows). This might be explained by a nonlinear signal response in the Orbitrap. While complex signal processing steps are implemented to address this issue†, a perfect linear relationship between MS2 reporter ion intensities and their corresponding MS1 precursor intensities might not be achieved in all cases. Consequently, our model had to be adjusted. We substituted the total intensity in the isolation window with the total intensity of peptide fragment ions, which is calculated as the total ion current of an MS2 scan minus the total reporter ion intensity of the same scan. We termed this metric “total peptide ion current” or PIC in short. Notably, the relationship between PIC and the total reporter ion signal demonstrated a robust linear relation (supplemental Fig. S6, *A* and *B*, lower rows). Nevertheless, MS2 scans also fail to reveal the full extent of low-intensity signals contributing to the noise. Therefore, an updated model based on MS2 information still has to account for interference to faithfully predict the total reporter ion signal. Ultimately, we arrived at a multiple linear regression model formula that comprehensively describes the total reporter ion signal in MS2 scans (Equation 8), making it applicable to any MS2-quantified multiplex proteomics experiment.

Fitting this model to the yeast-human PSM data resulted in continuously improved prediction results with increasing numbers of relevant predictor variables (Fig. 4, *E*–*H*). Due to the strong predictive performance of the final model (Fig. 4*H*), we were able to determine the individual contributions of each model term to the overall predicted reporter ion signal. Independent model fitting (on the six lower complexity samples) revealed that about 20 percent of the total reporter ion signal originates from ion interference (supplemental Fig. S7). Approximately half of this signal (about 10% in total) could be attributed to noise, that is, interfering ions that are not directly visible in the MS1 isolation window range. To gain further insight, we repeated this analysis for different intensity bins of the data. We grouped PSMs into four equal-sized bins based on quartiles of precursor ion intensities (Q1-Q4) and performed separate model fitting to the first bin (x ≤ Q1) as well as the last bin (x > Q3) (supplemental Fig. S8, *A* and *B*) which are expected to be most dissimilar in their susceptibility to interference. For each full model fit, we again calculated the relative contribution of the individual model terms to the total predicted reporter ion signal. The results revealed that most of the reporter ion signal in the high intensity bin (about 90%) could already be explained by the precursor intensity in the MS1 isolation window (supplemental Fig. S8*C*). On the other hand, for the low intensity bin, this number was just 64%, and about 25% of the reporter ion signal was attributed to noise (supplemental Fig. S8*C*).

**Fig. 5:**
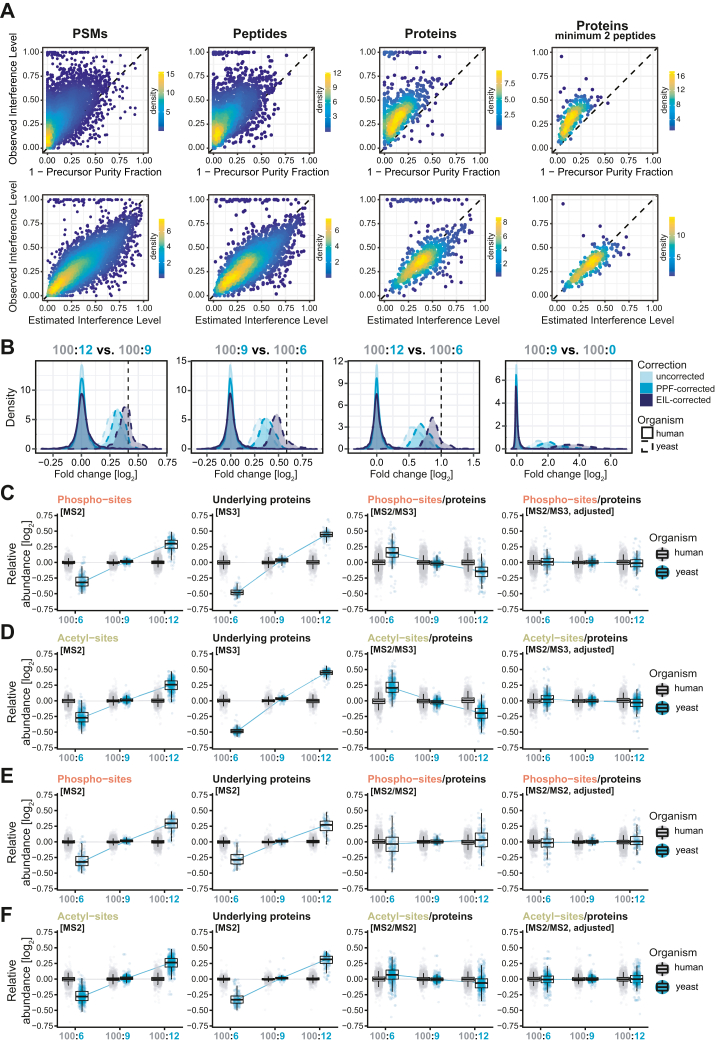
**Calculating and utilizing accurate interference estimates**. *A*, relationship between observed interference levels (OIL) and MS1 isolation window impurities (1-PPF) (*top* row) or estimated interference levels (EIL) (*bottom* row). Columns reflect distinct levels of yeast feature aggregation (PSMs, peptides, proteins, and proteins filtered for at least two unique and unambiguous peptides). OIL values were calculated based on feature-wise reporter ion intensities which were aggregated by summation. EIL and PPF values were aggregated by calculating weighted averages of PSM-specific EIL and PPF values, with weights reflecting the intensity contribution of individual PSMs to the aggregated reporter ion intensity. *B*, distribution of log_2_-transformed fold changes of yeast and human proteins, resulting from the pairwise group comparisons 100:12 *versus* 100:9, 100:9 *versus* 100:6, 100:12 *versus* 100:6, and 100:9 *versus* 100:0 (*left* to *right*). The *dashed black line* in each plot marks the expected theoretical fold change for yeast proteins and constitutes 1.33, 1.5, 2.0, and infinite, respectively. All proteins were additionally filtered for at least two unique and unambiguous peptides to reduce the potential for misspecification of human peptides as yeast. *C*–*F*, results of PTM site-to-protein normalization. Rows correspond to independent normalization results of MS2-quantified phospho or acetyl-sites, normalized to either MS3-quantified proteins (C-D) or MS2-quantified proteins (E-F). Columns show data before and after normalization. Each data point corresponds to a single yeast or human feature’s relative abundance in the respective group after log_2_ transformation and within-group averaging. PPF, precursor purity fraction; PTM, post-translational modification

It is noteworthy that this “global” prediction strategy can also be applied at the level of individual PSMs. This means that we could readily assess the relative contribution of reporter ion interference to the reporter ion signal for each individual PSM in the data by using the calculated model parameter estimates. We coined this model-based prediction of the fraction of reporter ion interference at MS2 level the “estimated interference level” or EIL (Equation 9), which can be interpreted as another ion purity metric like the previously introduced PPF. Yet unlike metrics for MS1 isolation window purity, EIL accurately estimates ion interference at MS2 level, with higher precision when aggregated from PSMs to peptides and proteins (Fig. 5*A*).

To validate the general applicability of model-based estimation of interference in multiplex proteomics, we applied our modeling strategy to other datasets (supplemental Fig. S9). First, we selected published datasets (51, 52) of MS2-quantified TKO9 and TKO11 proteomic standards (51). These commercial TMT-based standards each comprise three multiplexed congenic yeast KO strain proteomes which allow direct observation of interference for peptides of the missing proteins. Paolo *et al.* (51) took advantage of this property to calculate the so-called “interference-free index” (IFI), which translates to 1-OIL in our study. We compared OIL to model-based EIL values of the three knocked-out proteins and found a much-improved prediction of reporter ion interference over conventional purity metrics (supplemental Fig. S9, *A* and *B*). Second, we applied the modeling procedure to a dataset we recorded with FAIMS-MS2. Since FAIMS partially reduces interference (Fig. 2*C*), we were interested whether EIL can also faithfully predict the diminished interference signal. Indeed, we observed strongly improved prediction by this metric (supplemental Fig. S9*C*). Finally, we tested our approach on samples with an expected lower level of interference combined with uncommon peptide characteristics, namely samples enriched for lysine-acetylated or phosphorylated peptides from the yeast-human ground-truth experiment. Note that the decision tree algorithm introduced above (Fig. 4, *C* and *D*) is also able to consider PTM-specific effects on the fragmentation behavior. Again, the results emphasized the robustness and accuracy of EIL-based prediction of reporter ion interference and the advantage over conventional purity metrics, in PTM-enriched samples (supplemental Fig. S9, *D* and *E*).

### Interference Correction

Equipped with accurate estimates of reporter ion interference, we aimed to effectively correct ratio compression in any dataset. Savitski *et al.* calculated interference-corrected reporter ion intensities (23) using a conventional MS1-based purity estimate (termed “signal-to-interference” or S2I) (24). We adapted this approach by utilizing model-based EIL values instead, along with minor changes that aim to mitigate potential over-correction (Equations 10 and 11). We evaluated our algorithm's performance based on theoretical fold changes of 1.33, 1.5, 2.0, and infinite by comparing yeast protein signals across different groups in our two-proteome experiment (Fig. 1*A*). Conversely, human proteins had uniform relative abundances across all groups, thus mimicking proteins of unchanged expression levels. We compared three different modes of correction: No interference correction; correction based on the conventional MS1 isolation window purity metric PPF; and correction based on the estimated interference level EIL. The results highlight several key aspects. First, there is a notable improvement in fold change accuracy compared to the original correction approach (Fig. 5*B*). Second, EIL-based interference correction considerably decreased—but did not completely remove—ratio compression (Fig. 5*B*). Regarding this second point, we speculate that EIL-based interference correction has the potential to completely decompress fold changes; after all, EIL values manage to accurately estimate interference at MS2 level (Fig. 5*A* and supplemental Fig. S9). However, in the dataset presented here, this might have been prevented by a faint background of yeast peptide-derived interference. Since different amounts of yeast peptides were used in our experiment (Fig. 1), such an interference contribution would be *non-uniform*. This could have biased the between-sample normalization, ultimately diminishing group differences. Additionally, due to the applied ceiling of 0.8 for EIL during interference correction (Equation 11), a slight degree of ratio compression might have remained. Third, while the accuracy of fold change estimates increased, the correction procedure resulted in a loss in precision, as evidenced by the increased spread in human fold changes after correction (Fig. 5*B*). Notably, this tradeoff between accuracy and precision matches the already observed tradeoff that a change in interference entails (Fig. 2 and supplemental Fig. S2), yet here it is induced entirely computationally. Differential expression testing and ROC analyses on interference-corrected and uncorrected protein intensities shed further light on this tradeoff between accuracy and precision (supplemental Fig. S10). The separation quality between yeast and human protein populations in volcano plots was seemingly unaltered by the correction (supplemental Fig. S10, *A*–*D*). Corresponding ROC analyses (supplemental Fig. S10, *E*–*H*) corroborate that any differences in classification (based on *p*-values) are extremely minor, despite the notable changes in accuracy and precision. In other words, interference correction did not change the percentage of proteins correctly classified as differentially expressed, yet it improved the accuracy of fold change estimates (*i.e.* removed bias) but at the cost of higher fold change variance in the non-DE protein population.

### PTM Site-To-Protein Normalization

Multiplex proteomics is particularly well-suited for the study of posttranslational modifications, thanks to the joint processing of samples in one pool and the low number of missing values per labeling set. However, quantitative data on the PTM level is inherently confounded by the overall changes in underlying protein abundance (56). In order to accurately interpret results, differential abundances in modified peptides must be calibrated to protein levels, which can be assessed by quantifying unmodified peptides of the same proteins. This approach enables inference of changes on site level that are independent of changes on protein level, reflecting alterations in the posttranslational modification status (56, 57). Ideally, a simple normalization strategy, such as dividing site intensities by corresponding protein intensities or site fold changes by corresponding protein fold changes, can reveal this crucial information. However, in multiplex proteomics, this operation is complicated by ion interference, which compresses relative group differences on site and protein level independently. This introduces a risk for bias in site-to-protein normalized abundances or site-to-protein normalized fold changes, potentially leading to an incorrect reflection of biology.

Using our artificial yeast-human samples as a system with known ground-truth, we investigated this problem in more detail. MS2-quantified yeast and human phospho- and acetyl-site intensities were calibrated to both MS2 as well as FAIMS-MS3 RTS quantified protein signatures by arithmetic division (Fig. 5, *C*–*F*, column 1–3). In theory, existing group differences in yeast site abundances should level out after normalization to yeast proteins known to exhibit the same relative abundance pattern. Instead, the calculated ratios suggested varying acetylation and phosphorylation rates between groups (Fig. 5, *C*–*F*, column 3). For example, yeast sites calibrated to MS3-quantified proteins revealed a trend that is the exact opposite of the original yeast abundance pattern (Fig. 5, *C* and *D*, column 3). This highlights the danger of over-calibration when normalizing interference-affected site intensities to MS3-quantified protein intensities with little to no interference. On the other hand, calibrating to MS2-quantified proteins produced ratios that were more accurate on average but displayed large variation (Fig. 5, *E* and *F*, column 3). This observation underscores that similar overall levels of ratio compression are not sufficient to ensure unbiased normalization results, as individual site and protein pairs can still differ in their respective interference levels.

In light of these results, there is a need for unbiased site-to-protein normalization. Since the bias directly stems from unequal degrees of interference in individual site and protein pairs, we reasoned that adjusting for this disparity prior to ratio-building would remove this bias. We thus leveraged our model-based EIL values to establish such an algorithm (Equations (12), (13), (14), (15), (16), (17)). In brief, for each site and protein feature pair, reporter ion interference was artificially added to the feature with lower EIL until reaching equal levels. This adjustment only minimally affects the quantitative data before ratio-building and further does not increase within-group variances, as opposed to the interference correction approach described above. Following this adjustment to reach equal interference levels, normalization was performed as before *via* division. The resulting site-to-protein normalized abundances managed to accurately reflect the expected absence of between-group changes in site-to-protein ratios (Fig. 5, *C*–*F*, column 4). Moreover, the ratios displayed a much-reduced spread, particularly when normalized to MS2-quantified proteins (Fig. 5, *E* and *F*, column 4). Given these results, we think that this revised normalization approach promises effective reduction of bias from ion interference, which clears the way for the discovery of real biological changes at the posttranslational level through isobaric labeling-based quantification.

## Discussion

Despite extensive research into reporter ion interference in multiplex proteomics (https://pwilmart.github.io/blog/2020/01/05/TMT-ratio-distortions) (5, 6, 7, 8, 9, 10, 11, 12, 22, 23, 25, 26, 27, 28, 29, 30, 51, 52, 55, 58, 59, 60), a comprehensive mechanistic explanation of this phenomenon remained difficult. We attribute this challenge to two primary factors. Firstly, as suggested before (23), our study reveals that a significant portion of the signal associated with ion interference is not directly observable due to its removal during signal processing in Orbitrap instruments. Secondly, several factors influencing interference need to be considered when trying to understand, quantify, and mitigate interference. Consequently, previous efforts to correct ratio-compressed TMT data have primarily employed a phenomenological approach, relying on single measured variables to gauge the extent of ratio compression (6, 8, 9, 23, 25, 26, 55). However, since these single variables are not sufficient to fully account for the actual interference, the effectiveness of proposed computational methods has been limited. In contrast, technical solutions such as MS3 methods or charge state reduction (12, 13, 14, 16) have demonstrated more promising outcomes. Nevertheless, these alternatives rely on specific hardware requirements and introduce trade-offs concerning acquisition speed and signal strength. In our study, we aimed to address ratio compression by developing a holistic understanding, combining various approaches to determine the underlying causes of ion interference. We utilized a specially designed experimental system to directly assess the degree of interference and ultimately employed an exploratory computational model to identify and quantify the factors contributing to interference.

Our findings revealed that, on average, approximately 20% of the reporter ion signal was attributed to interference, a range similar to the findings reported by Wenger *et al.* who reported around 32% using a wider isolation window (12). These results highlight the difficulty of achieving accurate relative quantification in multiplex proteomics experiments and emphasize the need for appropriate correction measures. In addition, we investigated the influence of various parameters on the observed interference level and how they affect differential expression testing. These parameters included sample complexity, isolation window width, quantification method, maximum injection time, injection amount, and HPLC gradient length, and the results from this extensive screening are consistent with previously published reports (6, 7, 8, 9, 10, 13, 14, 16, 26, 51, 55). However, our analysis reveals that these parameters cannot be considered separately, since there are trade-offs between identification rate, quantitative accuracy and precision, and statistical power in DE analysis. Hence, multiparameter optimization to reach high identification rates, accurate fold change measurements, and correct classification of differentially expressed entities at the same time is a complex task. Our findings provide a guideline for selecting and optimizing the most relevant parameters in TMT multiplexing experiments.

The main goal of this study, however, was to perform a comprehensive characterization of interference to determine its cause and ultimately correct it with the use of computational models. Our analyses clearly show that interference is caused by TMT-labeled peptides or their putative fragments. Quenched TMT-label or its side-products are negligible. Moreover, our modeling approach shows that peptide ion parameters such as charge, amino acid composition, and number of bound TMT labels are important determinants of reporter ion generation that need to be considered in interference correction. A key finding, however, was that a major contribution to interference is caused by ions that are hidden in the noise of the spectrum. As a consequence, estimates of precursor purity based on the MS1 spectrum, such as PIF (43) or similar (24, 35, 38), are poor measures of interference and not ideal for interference correction, especially when noise levels are high (23). It was therefore necessary to introduce a novel metric for estimating ion interference, the model-based “EIL” which considers both visible and hidden non-precursor ion contribution to the reporter ion signal. Whether the “hidden interference” derives mainly from labeled peptides or from peptide fragments produced during ionization, as suggested by Erickson *et al.* (61) cannot be answered from our data. Most likely both factors contribute, although previous observations suggesting that interfering ions are mostly singly charged indicate that the contribution of fragments might be substantial (12). This would also fit our observation that the interference signal must be rather low and uniform to be fully removed during “de-noising” in the course of spectrum processing.

Building on our comprehensive understanding of the underlying mechanisms of interference, we adjusted our computational model to ensure broad applicability across all TMT datasets. A major consequence of this strategy was the computability of the EIL metric, which provides an accurate estimate of the degree of interference at PSM level. Our analysis convincingly demonstrated the effectiveness of EIL in correcting for ratio compression on a global scale. With our approach, we achieved higher quantitative accuracy with similar statistical power in differential expression analysis when compared to analyses using uncorrected data. As a result, we developed a software tool that enables correction of ratio compression that can be applied to any MS2-quantified TMT experiment (https://github.com/maxperutzlabs-ms/InterferenceModeling_in_MultiplexProteomics), as it is trained directly on each new dataset.

We further benchmarked our interference estimation strategy by testing its effectiveness for PTM datasets. Calibrating relative abundances of modified peptides to the according protein level is crucial for the assessment of changes in PTM patterns under different experimental conditions. However, PTM and protein information suffer from different degrees of compression, which often results in quantification artifacts when simply dividing site intensities by corresponding protein intensities. Our approach offers a solution to this problem, and we demonstrate its effectiveness in two scenarios: when PTM and protein information are both determined using MS2 measurements and when PTM information is obtained through MS2 while protein information is obtained through MS3 measurements. We have made all the required code available as documented repositories on GitHub to enable the community to apply our method (https://github.com/maxperutzlabs-ms/InterferenceModeling_in_MultiplexProteomics; https://github.com/maxperutzlabs-ms/SiteToProteinNormalization_in_MultiplexProteomics).

Finally, there are important assumptions and limitations that need to be considered. Firstly, in our calculation of the “PPF,” we assumed that the isolation efficiency is uniform across the whole isolation window, even though it is non-uniform (62) and even varies between instruments. However, given the relatively small deviation from uniformity, we do not anticipate a significant impact on our modeling approach. Alternatively, one could experimentally determine the isolation efficiency for a given instrument or infer it from the data to refine the calculation. Moreover, PPF might be further improved by accounting for detected nonprecursor isotope clusters in or around the isolation window, which offers additional information on the issue of unwanted co-isolation. Secondly, we assumed that every ion peak at MS1 level corresponds to a labeled peptide ion, disregarding the possibility of singly charged, non-labeled, non-peptide ion contamination. To address this, one could computationally remove singly charged signals or use fractionation/FAIMS to remove contamination if necessary. Furthermore, isobaric labeling experiments are mostly performed on Orbitrap FT-mass spectrometers due to the required instrument resolution for multiplexing. However, in the future, other mass spectrometers, such as TOF instruments, might be used that differ in signal detection and processing methods (63), which could alter the scan-specific information available for modeling ion interference. A similar circumstance could arise if Thermo Fisher changes scan-processing in Orbitrap instruments. Thus, the modeling strategy might need to be adapted accordingly. Another limitation that needs to be considered is the assumption that most proteins in the experiment have similar expression levels, leading to interference affecting all reporter channels evenly. While this assumption is generally valid, it may not always hold true. Therefore, when significant differences exist between multiplexed samples, an alternative correction strategy involving the filtering and/or differential weighting of individual proteins' PSMs based on measurement accuracy confidence, such as ion purity metrics calculated from the isolation window, may be more appropriate. Such strategies have been previously discussed and implemented by others (23, 28, 58) and we think that the EIL metric presented in this study can enhance their performance. However, it is important to note that strict filtering of PSMs will always result in a loss of identification numbers.

In summary, our study offers valuable insights into the nature of ion interference, as well as guidance on minimizing interference and optimizing DE testing in multiplex proteomics data. We believe that our work enhances the overall understanding of this fundamental problem and provides new computational strategies for effectively coping with ion interference and ratio compression.

## Data Availability

The mass spectrometry proteomics data (Thermo raw files and search results) have been deposited to the ProteomeXchange Consortium *via* the PRIDE (64) partner repository with the dataset identifier PXD040449. A detailed summary of all measurement raw files and their contribution to the individual results of this study is listed in supplemental Table S2. Relevant protein and PTM identification tables are available as supplemental Tables S3–S5. All code used in the analysis is available on GitHub (https://github.com/maxperutzlabs-ms/Publication_Resources). Additionally, we have created two documented GitHub repositories that provide the computational and statistical methods presented in this study in the form of ready-to-use R scripts (https://github.com/maxperutzlabs-ms/InterferenceModeling_in_MultiplexProteomics; https://github.com/maxperutzlabs-ms/SiteToProteinNormalization_in_MultiplexProteomics).

## Supplemental data

This article contains supplemental data.

## Conflict of interest

The authors declare no competing interests.

## Supplementary data

Madern et al_SupplementalMaterial

## Footnotes

† Personal communication with Thermo Fisher Scientific.
