# Supplementary material for: A Causal Model of Ion Interference Enables Assessment and Correction of Ratio Compression in Multiplex Proteomics
Source: Mol Cell Proteomics. 2023 Dec 12;23(1):100694. doi: 10.1016/j.mcpro.2023.100694 (PMC10828822; doi:10.1016/j.mcpro.2023.100694)
Supplement: Madern et al_SupplementalMaterial [file mmc1.pdf]

A Causal Model of Ion Interference Enables Assessment and Correction of Ratio Compression in Multiplex Proteomics

Moritz Madern, Wolfgang Reiter, Florian Stanek, Natascha Hartl, Karl Mechtler, Markus Hartl

Included Supplemental Material

Figure S1 ………………………………………………………………………………………….. S-2

Figure S2 ………………………………………………………………………………………….. S-3

Figure S3 ………………………………………………………………………………………….. S-5

Figure S4 ………………………………………………………………………………………….. S-6

Figure S5 ………………………………………………………………………………………….. S-7

Figure S6 ………………………………………………………………………………………….. S-8

Figure S7 ………………………………………………………………………………………….. S-10

Figure S8 ………………………………………………………………………………………….. S-11

Figure S9 ………………………………………………………………………………………….. S-13

Figure S10 ………………………………………………………………………………………… S-15

Table S1 …………………………………………………………………………………………….. S-17

Table S2 …………………………………………………………………………………………….. S-18

Figure S1

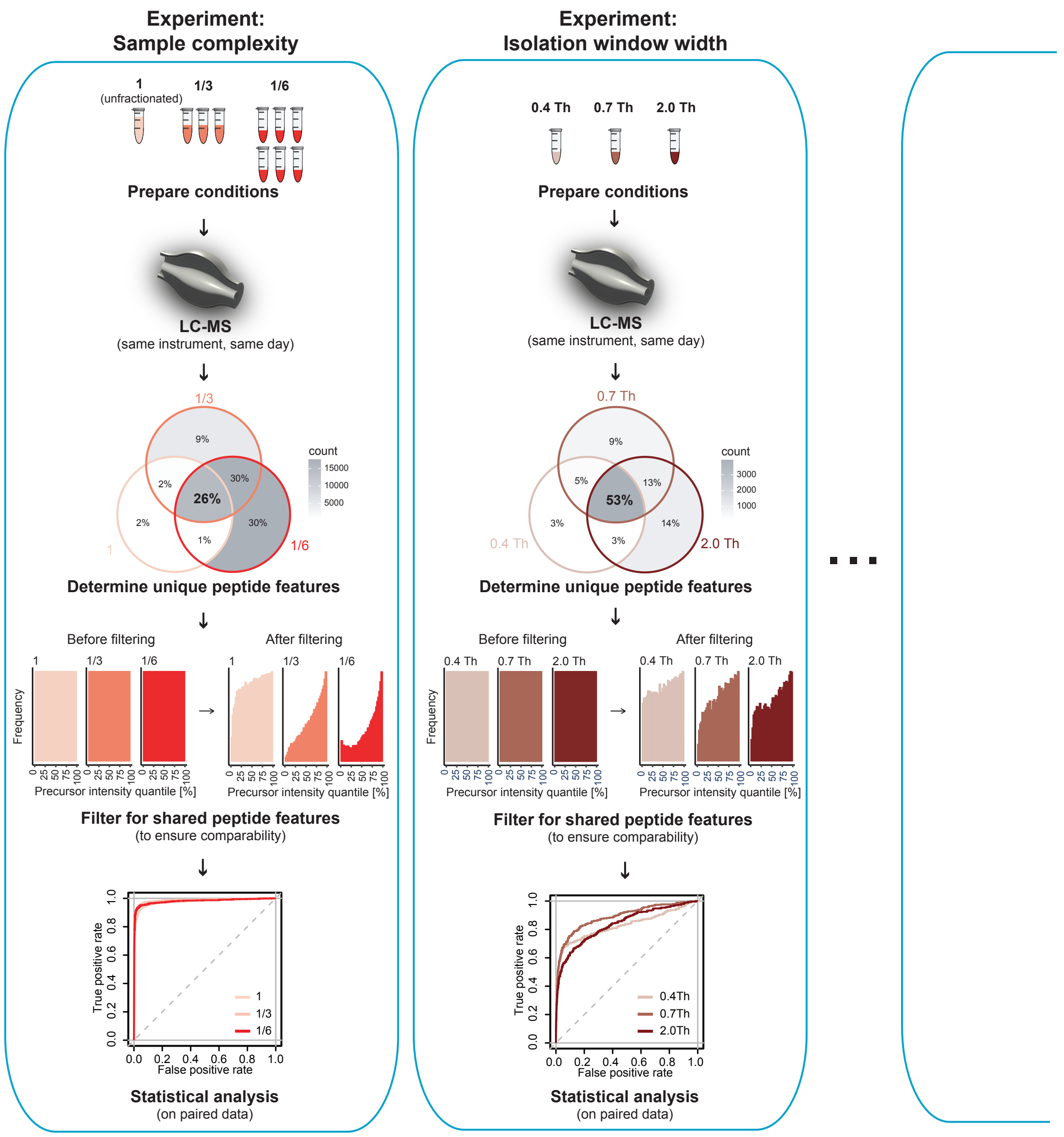

Figure S1 – Analysis strategy for systematic exploration of measurement parameters. Panels depict the entire experimental and computational workflow for comparative analyses of varying sample complexities (left) and varying isolation window widths (right). Workflow for the other experiments (exploring varying quantification strategies, maximum injection times, injection amounts and gradient lengths) are omitted but follow the same pattern.

Figure S2

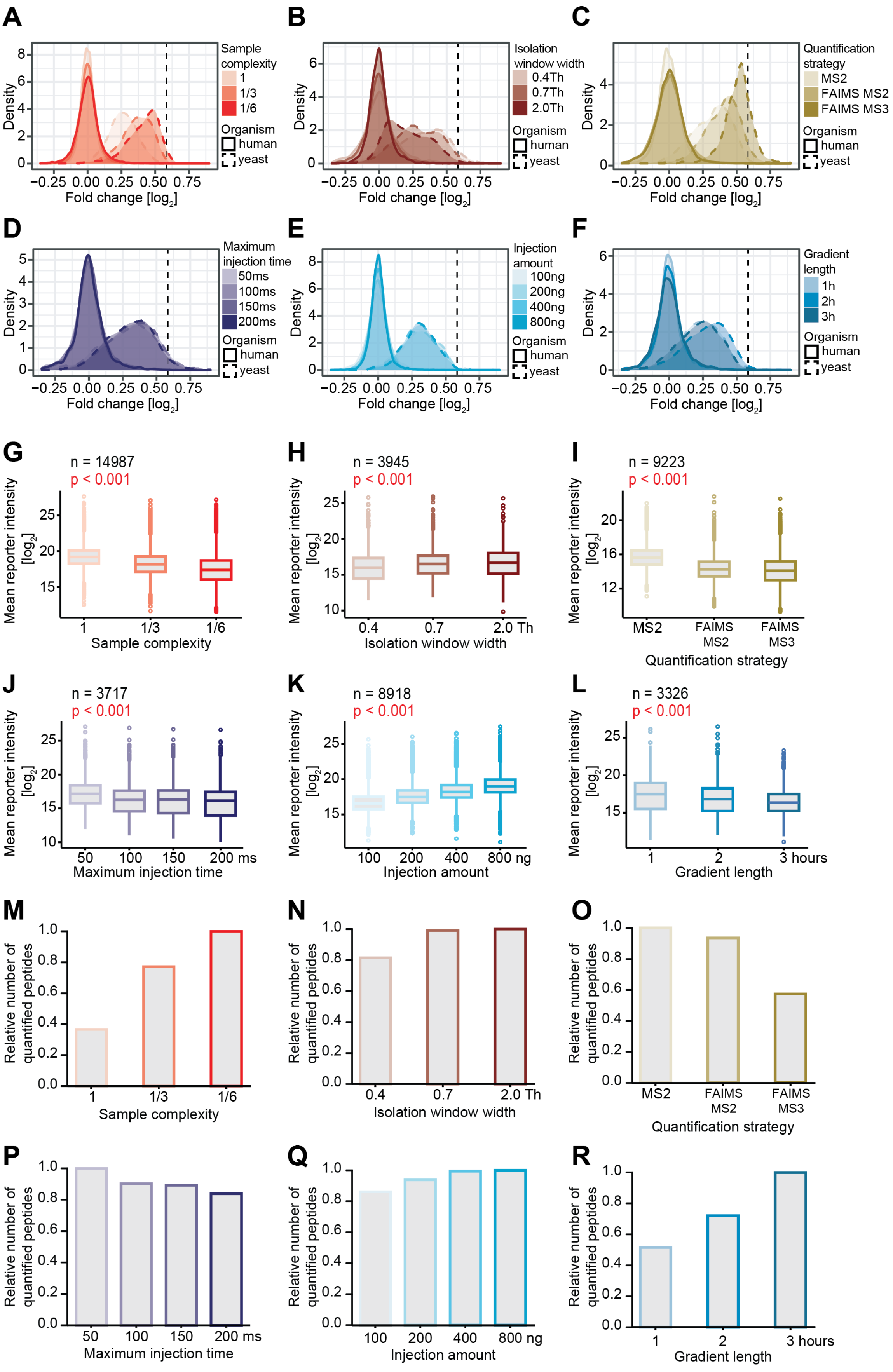
A
B
C
D
E
F
G
H
I
J
K
L
M
N
O
P
Q
R
Density
Fold change [log2]
Sample complexity
Isolation window width
Quantification strategy
Maximum injection time
Injection amount
Gradient length
Organism
human
yeast
MS2
FAIMS MS2
FAIMS MS3
50ms
100ms
150ms
200ms
100ng
200ng
400ng
800ng
1h
2h
3h
0.4Th
0.7Th
2.0Th
n = 14987
n = 3945
n = 9223
n = 3717
n = 8918
n = 3326
p < 0.001
Mean reporter intensity [log2]
Relative number of quantified peptides
1/3
1/6
2.0 Th
50 100 150 200 ms
100 200 400 800 ng
1 2 3 hours

Figure S2 – Supplement to Figure 2. (A-F) Distribution of log_2-transformed fold changes of yeast and human peptide features independently quantified in all conditions within a comparison, resulting from the comparison between groups 100:9 and 100:6. The dashed black line marks the theoretical fold change for yeast peptides. **(G-L)** Distribution of average reporter intensities of yeast and human peptide features independently quantified in all conditions within a comparison. P-values denote statistical significance for overall differences between conditions (Friedman test). **(M-R)** Relative number of unique peptides quantified per condition. The highest number in each comparison was scaled to 1.

Figure S3

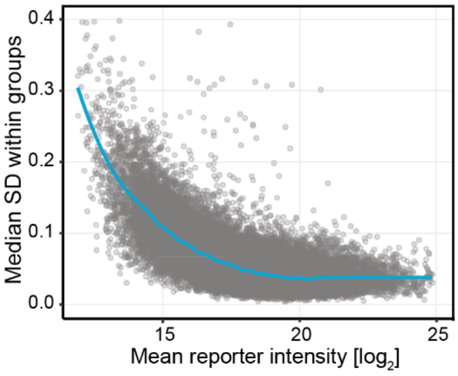

Figure S3 – Mean-variance trend in isobaric labeling-based quantification. Empirical mean-variance trend of log_2-transformed reporter ion intensities of MS2-quantified yeast and human peptides. The turquoise line represents a loess-fit with a span parameter of 0.05. Each data point corresponds to a single PSM.

Figure S4

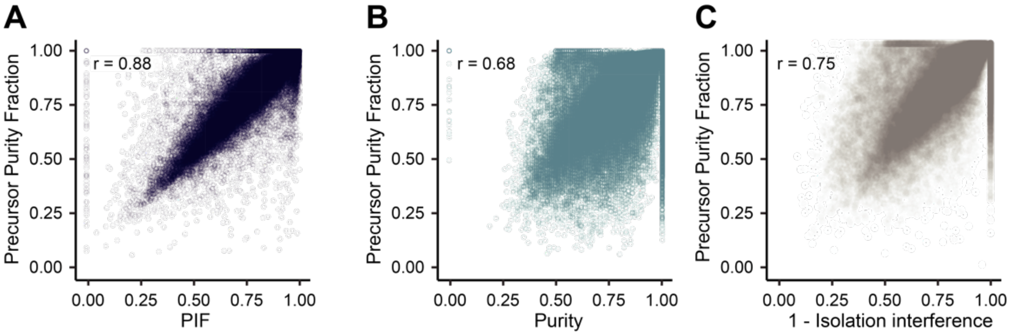

Figure S4 – Comparison of Precursor Purity Fraction (PPF) to existing purity metrics. Each data point corresponds to a single PSM. r denotes the calculated Pearson correlation coefficient. **(A)** Comparison with the "PIF" metric implemented in MaxQuant. **(B)** Comparison with the "Purity" metric implemented in FragPipe. **(C)** Comparison with the "Isolation Interference" metric implemented in the Proteome Discoverer software MS Amanda**.**

Figure S5

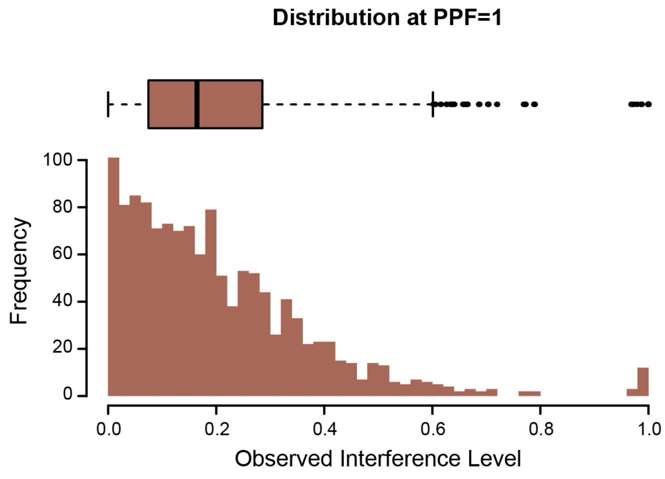

Figure S5 – Supplement to Figure 3A. Distribution of observed interference levels (OIL) of yeast PSMs at PPF=1. Note that only a small fraction of yeast PSMs actually appears free of reporter ion interference (i.e. OIL=0). The sharp rise in counts at OIL=1 is assumed to stem from human peptides that were misspecified by the search engine as yeast peptides at an FDR of 1% at PSM level.

Figure S6

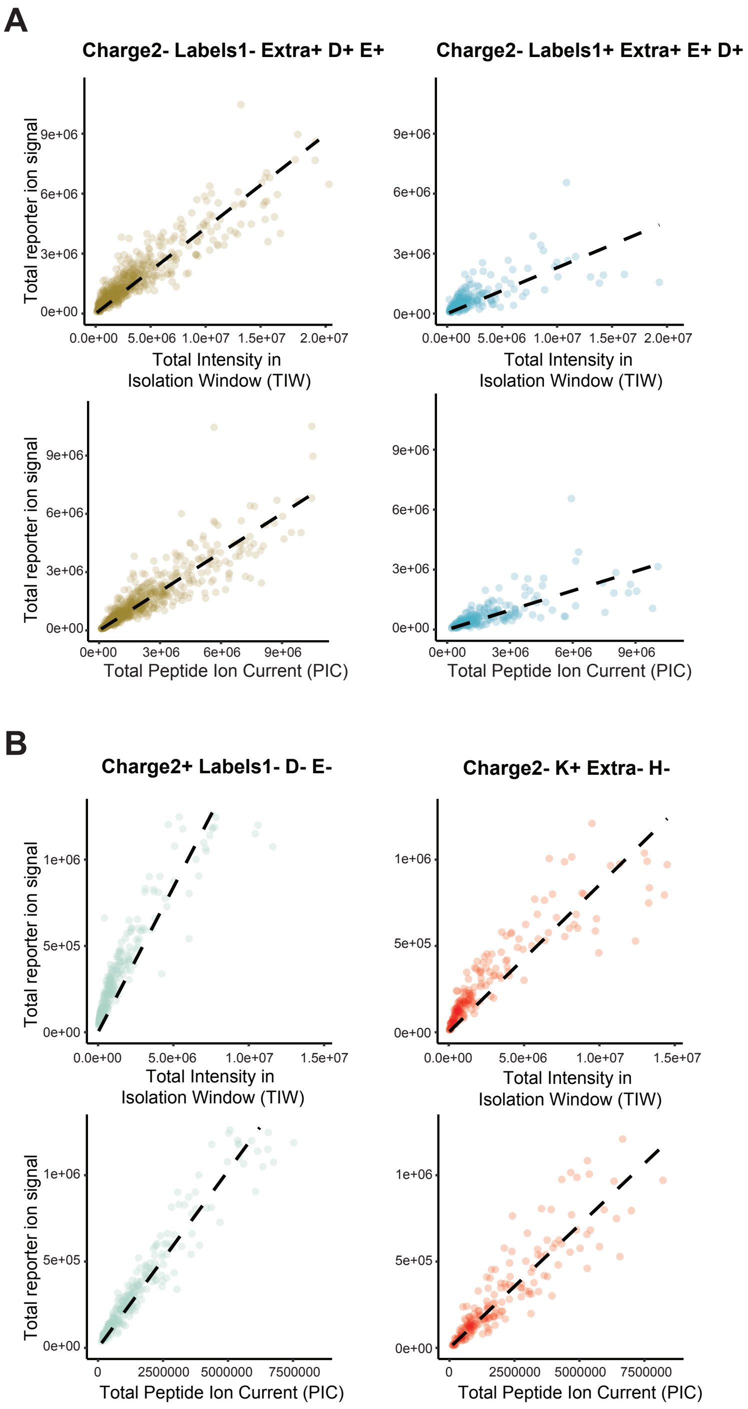
A
Charge2- Labels1- Extra+ D+ E+
Charge2- Labels1+ Extra+ E+ D+
Total reporter ion signal
Total Intensity in Isolation Window (TIW)
Total Peptide Ion Current (PIC)
B
Charge2+ Labels1- D- E-
Charge2- K+ Extra- H-
Total reporter ion signal
Total Intensity in Isolation Window (TIW)
Total Peptide Ion Current (PIC)

Figure S6 – Linearity in regression modeling. Dependence of the total reporter ion signal on the total intensity in the isolation window (TIW) (upper rows) and the total peptide ion current (PIC) (lower rows) for PSMs of two distinct empirical peptide classes per unique dataset. The dashed line in each plot reflects a simple linear regression fit. **(A)** Data showing two example peptide classes in the yeast-human mixture dataset (raw file "20201030_[…]_complexity_P1"). **(B)** Data showing two example peptide classes in the TKO9 dataset published with the original publication (PMID: 27400695).

Figure S7

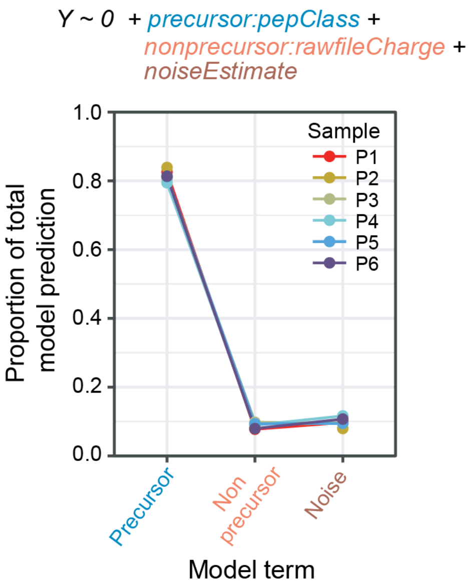

Figure S7 – Partition of model prediction. Results display model term-specific proportions of predicted reporter ion signal with respect to total predicted reporter ion signal across all PSMs of a single measurement run. The model was fit separately for each of the six samples of reduced sample complexity (P1-P6) measured via MS2-based quantification. The three model terms (x-axis) relate to different parts of the model equation, as coded by color. The second term (Non precursor) and the third term (Noise) denote the visible and the invisible contribution of ion interference, respectively, to the total reporter ion signal at MS2 level.

Figure S8

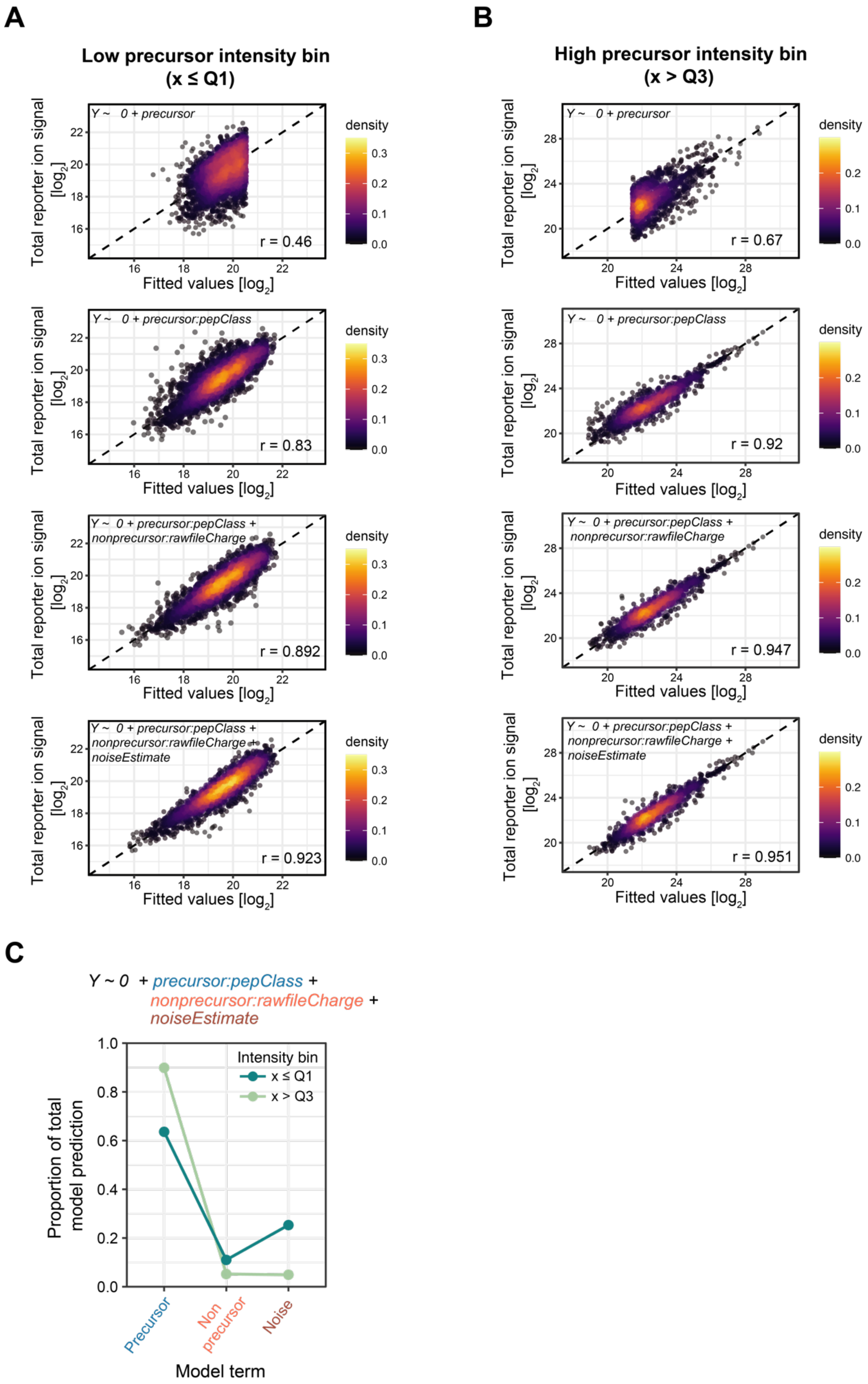
A
Low precursor intensity bin
(x ≤ Q1)
B
High precursor intensity bin
(x > Q3)
Y ~ 0 + precursor
Y ~ 0 + precursor:pepClass
Y ~ 0 + precursor:pepClass + nonprecursor:rawfileCharge
Y ~ 0 + precursor:pepClass + nonprecursor:rawfileCharge + noiseEstimate
Total reporter ion signal [log2]
Fitted values [log2]
density
r = 0.46
r = 0.83
r = 0.892
r = 0.923
r = 0.67
r = 0.92
r = 0.947
r = 0.951
C
Y ~ 0 + precursor:pepClass + nonprecursor:rawfileCharge + noiseEstimate
Intensity bin
x ≤ Q1
x > Q3
Proportion of total model prediction
Precursor
Non precursor
Noise
Model term

Figure S8 – Modeling total reporter ion signal for low and high precursor intensity bins. (A) Model prediction of the measured total reporter ion signal for the low precursor intensity bin ($x \leq Q1$) by four nested linear regression models. Model formulas are depicted at the top of each plot. Each data point corresponds to a single yeast or human PSM from raw file "20201030_[…]_complexity_P1". Data points are colored by point density. The dashed line represents the identity function ($y = x$) and therefore reflects a perfect prediction. An increasing number of predictor variables were sequentially included into the model from top to bottom. r denotes the calculated Pearson correlation coefficient. **(B)** The same as in (A) but for the high precursor intensity bin ($x > Q3$). **(C)** Model term-specific proportions of predicted reporter ion signal with respect to total predicted reporter ion signal across all PSMs of low ($x \leq Q1$) and high ($x > Q3$) precursor intensity bins, quantified in samples of reduced sample complexity (P1-P6). The three model terms (x-axis) relate to different parts of the model equation, as coded by color. The second term (Non precursor) and the third term (Noise) denote the visible and the invisible contribution of ion interference, respectively, to the total reporter ion signal at MS2 level.

Figure S9

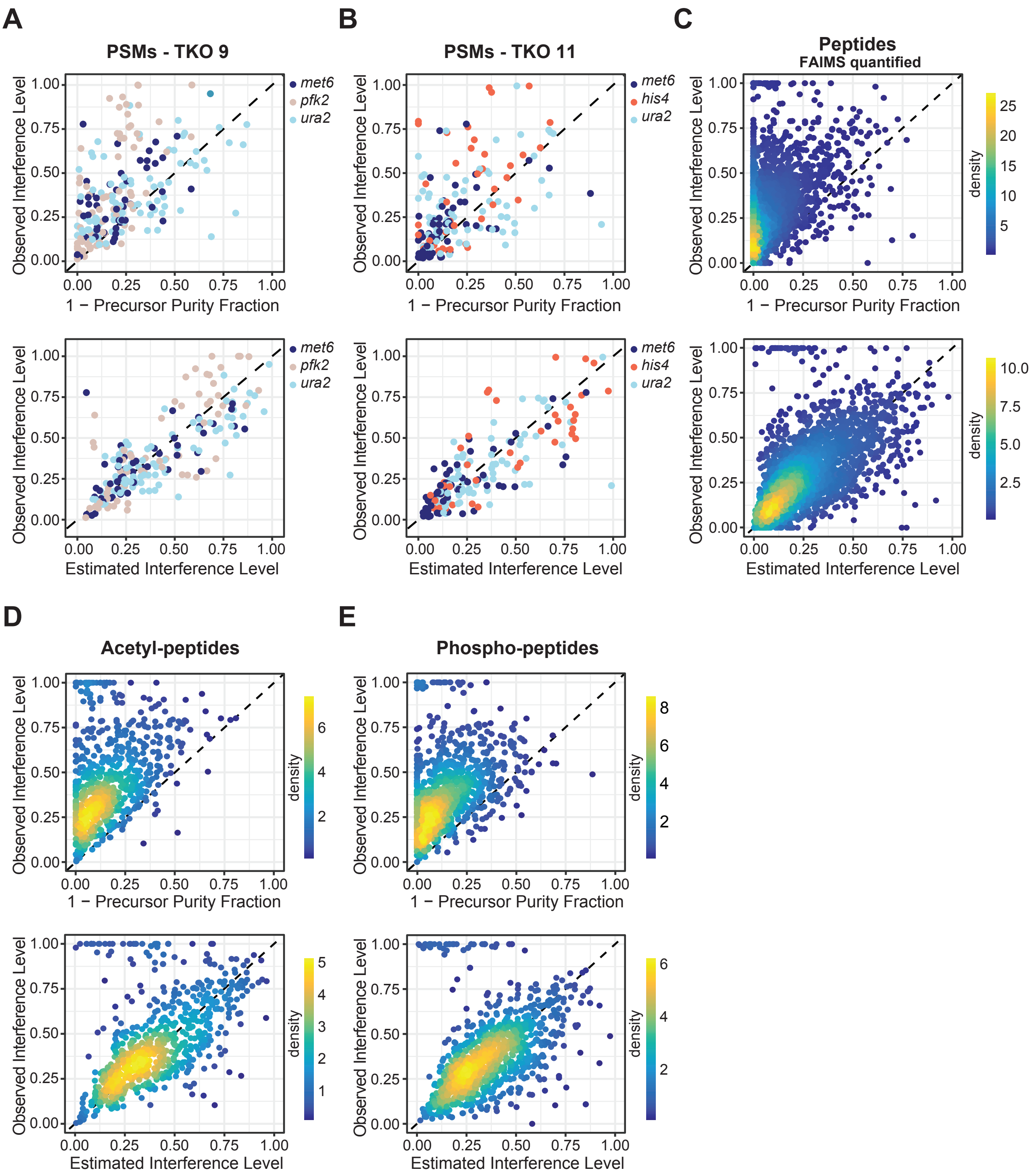

A
PSMs - TKO 9
B
PSMs - TKO 11
C
Peptides
FAIMS quantified
met6
pfk2
ura2
his4
Observed Interference Level
1 − Precursor Purity Fraction
Estimated Interference Level
density
D
Acetyl-peptides
E
Phospho-peptides

Figure S9 – Evaluation of EIL in other data sets. Plots show the relationship between observed interference levels (OIL) and MS1 ion impurities (1-PPF) (top row) or estimated interference levels (EIL) (bottom row). **(A)** PSM level data of previously published triplicate measurements of the TKO9 proteomics standard (PMID: 27400695). Observed interference levels (OIL) were determined for quantified peptides of the three KO genes met6, pfk2 and ura2. **(B)** PSM level data of previously published triplicate measurements of the TKO11 proteomics standard (PMID: 32202424). Observed interference levels were determined for quantified peptides of the three KO genes met6, his4 and ura2. **(C)** Peptide level data of the yeast-human mixture experiment using FAIMS-MS2 quantification. Observed interference levels (OIL) were determined for yeast peptides. Measurements are the same as shown in Figure 2C (FAIMS-MS2). **(D)** Acetyl-peptide level data of the yeast-human mixture experiment using MS2 quantification. Observed interference levels (OIL) were determined for acetylated yeast peptides. **(E)** Phospho-peptide level data of the yeast-human mixture experiment using MS2 quantification. Observed interference levels (OIL) were determined for phosphorylated yeast peptides.

Figure S10

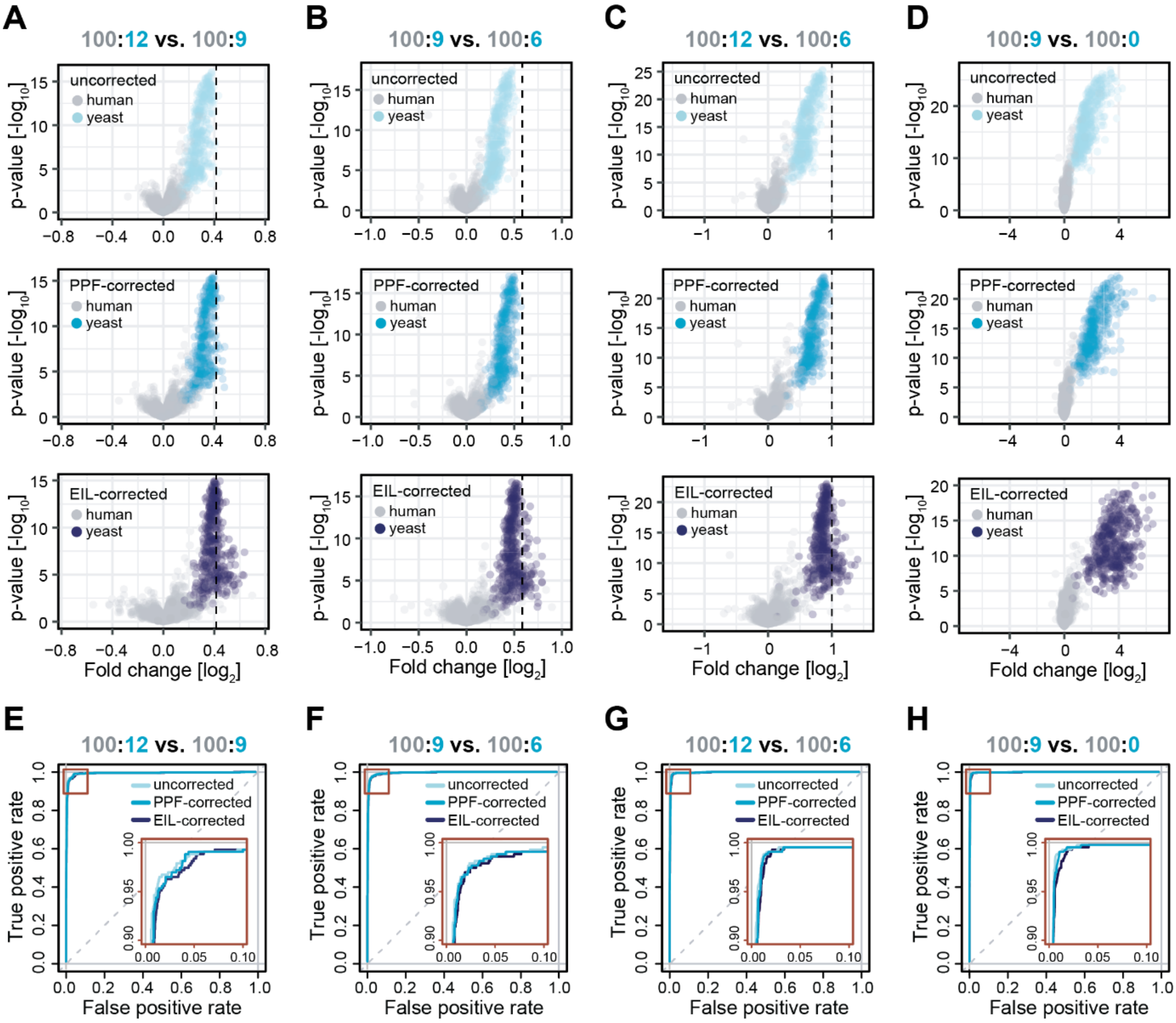
A
100:12 vs. 100:9
B
100:9 vs. 100:6
C
100:12 vs. 100:6
D
100:9 vs. 100:0
uncorrected
PPF-corrected
EIL-corrected
human
yeast
p-value [-log10]
Fold change [log2]
E
100:12 vs. 100:9
F
100:9 vs. 100:6
G
100:12 vs. 100:6
H
100:9 vs. 100:0
True positive rate
False positive rate

Figure S10 – Supplement to Figure 5B. (A-D) Volcano plots illustrating results of protein level DE testing and fold change accuracy and precision for three distinct interference correction strategies. Columns represent the different pairwise group comparisons already shown in Figure 5B: (A) 100:12 vs. 100:9; (B) 100:9 vs. 100:6; (C) 100:12 vs. 100:6; and (D) 100:9 vs. 100:0. The dashed black line in each plot marks the theoretical fold change for yeast proteins and constitutes (A) 1.33, (B) 1.5, (C) 2.0 and (D) infinite, respectively. Rows represent different interference corrections strategies: uncorrected, PPF-corrected and EIL-corrected (top to bottom). Each data point corresponds to a single yeast or human protein. All proteins were additionally filtered for at least 2 unique and unambiguous peptides to minimize potential misspecification of human as yeast and vice-versa. Statistical testing was performed via the Limma-trend testing procedure on $\log_2$-transformed protein intensities**.** Aggregation to protein intensities was performed by summation of individual PSM's normalized and interference-corrected reporter ion intensities. **(E-H)** ROC analyses based on the DE testing results. The true positive rate was calculated as the fraction of yeast proteins correctly classified as differentially expressed at a certain significance level. The false positive rate was calculated as the fraction of human proteins incorrectly classified as differentially expressed at a certain significance level.

Table S1

	General measurement settings									
	CVs	MS instrument	Number of samples	Gradient length	Cycle time	MS1 res	MS1 AGC target	MS1 maximum IT	Isolation window	Intensity threshold
MS2	-	Eclipse	2	2h	3s	120k	400000	50ms	0.7Th	25000
FAIMS-MS2	-40, -55, -70	Eclipse	2	2h	1.2s	60k	400000	50ms	0.7Th	25000
FAIMS-MS3 RTS	-40, -55, -70	Eclipse	2	2h	1.2s	120k	400000	50ms	0.7Th	10000

	MS2 scan settings					MS3 scan settings					
	MS2 detector type	MS2 AGC target	MS2 collision energy	MS2 Orbitrap resolution	MS2 maximum IT	MS3 detector type	MS3 AGC target	MS3 collision energy	MS3 Orbitrap resolutio n	MS3 maximum IT	MS3 Number of notches
MS2	Orbitrap	150000	HCD 34%	50k	100ms	-	-	-	-	-	-
FAIMS-MS2	Orbitrap	150000	HCD 34%	50k	100ms	-	-	-	-	-	-
FAIMS-MS3 RTS	IonTrap	10000	CID 30%	-	50ms	Orbitrap	150000	HCD 50%	50k	150ms	10

Table S1 – Supplement to Figure 2C, **2I** and **2O.** Summary of instrument parameters for comparative measurements using different quantification strategies. All measurements were performed on the same instrument (Eclipse) to ensure comparability.

Table S2

Experiment Name	Description	Raw files	Search Engine	Search Results Folder on PRIDE
Main Experiment: Interference Modeling in Yeast-Human Dataset	About interference modeling and interference-correction for data of yeast-human mixture samples of reduced sample complexity measured via MS2-based quantification. Comprises the majority of figures in the study: Figure 3, Figure 4, Figure 5A-B, Figure S3, Figure S4A, Figure S5, Figure S6A, Figure S7, Figure S8, Figure S10.	20201030_QExHFX1_RSLC1_Madern_Hartl_UW_MFPL__complexity_P1<br>20201030_QExHFX1_RSLC1_Madern_Hartl_UW_MFPL__complexity_P2<br>20201030_QExHFX1_RSLC1_Madern_Hartl_UW_MFPL__complexity_P3<br>20201030_QExHFX1_RSLC1_Madern_Hartl_UW_MFPL__complexity_P4<br>20201030_QExHFX1_RSLC1_Madern_Hartl_UW_MFPL__complexity_P5<br>20201030_QExHFX1_RSLC1_Madern_Hartl_UW_MFPL__complexity_P6	MaxQuant	MaxQuant_MainExperiment_txt
Comparative Measurements	About characterizing variations in sample- and measurement-specific parameters in their effect on ion interference, ratio compression, differential expression analysis and peptide numbers. Comprises the following figures: Figure 2, Figure S1, Figure S2, Table S1.	20201030_QExHFX1_RSLC1_Madern_Hartl_UW_MFPL__complexity_P1<br>20201030_QExHFX1_RSLC1_Madern_Hartl_UW_MFPL__complexity_P2<br>20201030_QExHFX1_RSLC1_Madern_Hartl_UW_MFPL__complexity_P3<br>20201030_QExHFX1_RSLC1_Madern_Hartl_UW_MFPL__complexity_P4<br>20201030_QExHFX1_RSLC1_Madern_Hartl_UW_MFPL__complexity_P5<br>20201030_QExHFX1_RSLC1_Madern_Hartl_UW_MFPL__complexity_P6<br>20201030_QExHFX1_RSLC1_Madern_Hartl_UW_MFPL__complexity_P1P4<br>20201030_QExHFX1_RSLC1_Madern_Hartl_UW_MFPL__complexity_P2P5<br>20201030_QExHFX1_RSLC1_Madern_Hartl_UW_MFPL__complexity_P3P6<br>20201030_QExHFX1_RSLC1_Madern_Hartl_UW_MFPL__complexity_P1P2P3P4P5P6<br>20210723_QExHFX2_LC4_Madern_Hartl_UW_MPL_TMT_16plex_TMT_gradient_IW_0p4<br>20210723_QExHFX2_LC4_Madern_Hartl_UW_MPL_TMT_16plex_TMT_gradient_IW_0p7<br>20210723_QExHFX2_LC4_Madern_Hartl_UW_MPL_TMT_16plex_TMT_gradient_IW_2p0<br>20210827_Eclipse_LC3_noFAIMS_PepMap50cm-precolumn_P2<br>20210827_Eclipse_LC3_noFAIMS_PepMap50cm-precolumn_P5<br>20210830_Eclipse_LC3_FAIMS_MS2_Madern_Hartl_UW_MPL_TMT_P2<br>20210830_Eclipse_LC3_FAIMS_MS2_Madern_Hartl_UW_MPL_TMT_P5<br>20210830_Eclipse_LC3_FAIMS_MS3_RTS_Madern_Hartl_UW_MPL_TMT_P2<br>20210830_Eclipse_LC3_FAIMS_MS3_RTS_Madern_Hartl_UW_MPL_TMT_P5<br>20210825_QExHFX2_LC4_Madern_Hartl_UW_MPL_TMT_50ms<br>20210907_QExHFX2_LC4_Madern_Hartl_UW_MPL__50ms_maxIT_p3<br>20210907_QExHFX2_LC4_Madern_Hartl_UW_MPL_TMT_p5_50ms<br>20210825_QExHFX2_LC4_Madern_Hartl_UW_MPL_TMT_100ms<br>20210907_QExHFX2_LC4_Madern_Hartl_UW_MPL__100ms_maxIT_p3<br>20210907_QExHFX2_LC4_Madern_Hartl_UW_MPL_TMT_p5_100ms<br>20210825_QExHFX2_LC4_Madern_Hartl_UW_MPL_TMT_150ms<br>20210907_QExHFX2_LC4_Madern_Hartl_UW_MPL__150ms_maxIT_p3<br>20210907_QExHFX2_LC4_Madern_Hartl_UW_MPL_TMT_p5_150ms<br>20210825_QExHFX2_LC4_Madern_Hartl_UW_MPL_TMT_200ms<br>20210907_QExHFX2_LC4_Madern_Hartl_UW_MPL__200ms_maxIT_p3<br>20210907_QExHFX2_LC4_Madern_Hartl_UW_MPL_TMT_p5_200ms<br>20210309_QExHFX1_RSLC1_Madern_Hartl_UW_MFPL__ProtP4_100ng<br>20210309_QExHFX1_RSLC1_Madern_Hartl_UW_MFPL__ProtP4_200ng<br>20210309_QExHFX1_RSLC1_Madern_Hartl_UW_MFPL__ProtP4_400ng<br>20210309_QExHFX1_RSLC1_Madern_Hartl_UW_MFPL__ProtP4_800ng<br>20210715_QExHFX2_RSLC4_Madern_Hartl_UW_MPL_TMT_grad1h<br>20210715_QExHFX2_RSLC4_Madern_Hartl_UW_MPL_TMT_grad2h<br>20210715_QExHFX2_RSLC4_Madern_Hartl_UW_MPL_TMT_grad3h	FragPipe	Fragpipe_ComparativeMeasurements
Interference Modeling in TKO9 Dataset	About interference modeling for data of TKO9 standard samples. Raw files correspond to previously published triplicate measurements of the TKO9 proteomics standard (PMID: 27400695). Raw files available at: https://www.ebi.ac.uk/pride/archive/projects/PXD008009. Comprises the following figures: Figure S6B, Figure S9A.	L00761<br>L00763<br>L00765	MaxQuant	MaxQuant_TKO9_TKO11_msms
Interference Modeling in TKO11 Dataset	About interference modeling for data of TKO11 standard samples. Raw files correspond to previously published triplicate measurements of the TKO11 proteomics standard (PMID: 32202424). Raw files available at: https://www.ebi.ac.uk/pride/archive/projects/PXD017803. Comprises the following figure: Figure S9B.	FigS2_qb03861_TKO11<br>FigS2_qb03862_TKO11<br>FigS2_qb03863_TKO11	MaxQuant	MaxQuant_TKO9_TKO11_msms
Interference Modeling in Yeast-Human Dataset	About interference modeling and interference-correction for data of yeast-human mixture samples of reduced sample complexity measured viaFAIMS MS2-based quantification. Comprises the following figure: Figure S9C.	20210830_Eclipse_LC3_FAIMS_MS2_Madern_Hartl_UW_MPL_TMT_P2<br>20210830_Eclipse_LC3_FAIMS_MS2_Madern_Hartl_UW_MPL_TMT_P5	MaxQuant	MaxQuant_FAIMS_MS2_txt
Comparison of Isolation Window Purity Metrics	About comparing "Precursor Purity Fraction" (PPF) with existing purity metrics from other software. These are: "PIF" from Maxquant, "Purity" from Fragpipe, "Isolation Interference" from Proteome Discoverer software MSAmanda. Comprises the following figure: Figure S4.	20201030_QExHFX1_RSLC1_Madern_Hartl_UW_MFPL__complexity_P1<br>20201030_QExHFX1_RSLC1_Madern_Hartl_UW_MFPL__complexity_P2<br>20201030_QExHFX1_RSLC1_Madern_Hartl_UW_MFPL__complexity_P3<br>20201030_QExHFX1_RSLC1_Madern_Hartl_UW_MFPL__complexity_P4<br>20201030_QExHFX1_RSLC1_Madern_Hartl_UW_MFPL__complexity_P5<br>20201030_QExHFX1_RSLC1_Madern_Hartl_UW_MFPL__complexity_P6	MaxQuant, FragPipe, Proteome Discoverer (MS Amanda)	MaxQuant_MainExperiment_txt,Fragpipe_ComparativeMeasurements, PD_MSAmanda_MS2
Site-To-Protein Normalization in Multiplex Proteomics	About Interference modeling and site-to-protein normalization for data of yeast-human mixture samples. Comprises the following figures: Figure 5C-F, Figure S9D-E.	20200909_QExHFX1_RSLC1_Madern_Hartl_UW_MFPL_master_exp_p1_acet<br>20200909_QExHFX1_RSLC1_Madern_Hartl_UW_MFPL_master_exp_p2_acet<br>20200909_QExHFX1_RSLC1_Madern_Hartl_UW_MFPL_master_exp_p3_acet<br>20200909_QExHFX1_RSLC1_Madern_Hartl_UW_MFPL_master_exp_p4_acet<br>20200909_QExHFX1_RSLC1_Madern_Hartl_UW_MFPL_master_exp_p5_acet<br>20200909_QExHFX1_RSLC1_Madern_Hartl_UW_MFPL_master_exp_p6_acet<br>20200909_QExHFX1_RSLC1_Madern_Hartl_UW_MFPL_master_exp_p1_phospho<br>20200909_QExHFX1_RSLC1_Madern_Hartl_UW_MFPL_master_exp_p2_phospho<br>20200909_QExHFX1_RSLC1_Madern_Hartl_UW_MFPL_master_exp_p3_phospho<br>20200909_QExHFX1_RSLC1_Madern_Hartl_UW_MFPL_master_exp_p4_phospho<br>20200909_QExHFX1_RSLC1_Madern_Hartl_UW_MFPL_master_exp_p5_phospho<br>20200909_QExHFX1_RSLC1_Madern_Hartl_UW_MFPL_master_exp_p6_phospho<br>20201030_QExHFX1_RSLC1_Madern_Hartl_UW_MFPL__complexity_P2<br>20201030_QExHFX1_RSLC1_Madern_Hartl_UW_MFPL__complexity_P5<br>20210830_Eclipse_LC3_FAIMS_MS3_RTS_Madern_Hartl_UW_MPL_TMT_P2<br>20210830_Eclipse_LC3_FAIMS_MS3_RTS_Madern_Hartl_UW_MPL_TMT_P5	MaxQuant	MaxQuant_SiteToProteinNorm_txt

Table S2 – Designation of MS raw files to experiments. Table presents a detailed overview of all measurement raw files and associated database search results (available on PRIDE) and their contribution to the individual results of this study.
